# Blocking AGEs reduces airway epithelial viral susceptibility in diabetes-exacerbated COPD

**DOI:** 10.1038/s44321-026-00502-9

**Published:** 2026-08-17

**Authors:** Zhenfeng Chen, Yueying Qin, Bingqi Lin, Linle Jiang, Zhencheng Feng, Shiying Chen, Zuodong Ye, Min Zhou, Jia Li, De Yun Wang, Nanshan Zhong, Panpan Hou, Ziqing Zhou, Lei Wang, Yang Peng

**Affiliations:** 1 State Key Laboratory of Respiratory Disease, National Clinical Research Center for Respiratory Disease, Guangzhou Institute of Respiratory Health, The First Affiliated Hospital, Guangzhou Medical University, Guangzhou, China; 2 Guangzhou National Laboratory, Guangzhou International Bio Island, Guangzhou, China; 3 Department of Allergy, The Third Affiliated Hospital of Sun Yat-sen University, Guangzhou, China; 4 Department of Otolaryngology, Infectious Diseases Translational Research Program, Yong Loo Lin School of Medicine, National University of Singapore, Singapore, Singapore

**Keywords:** Immunology, Metabolism, Respiratory System

## Abstract

Although diabetic complications are well-established in multiple organs, their impact on the respiratory system remains unclear. Clinical evidence indicates accelerated chronic obstructive pulmonary disease (COPD) progression in individuals with diabetes, yet the underlying mechanisms are poorly defined. Using single-cell RNA sequencing of airway epithelium, we identified distinct transcriptional alterations in COPD patients with or without diabetes, particularly in host–defense pathways related to viral infection, which is a key driver of COPD exacerbations. Functionally, hyperglycemia enhanced influenza virus susceptibility in both COPD-derived epithelial cells and COPD mice with diabetes, indicating compromised airway defense. We further demonstrated that hyperglycemia promotes advanced glycation end products (AGEs) accumulation in airway epithelium, with AGEs levels correlating with infection severity. Pharmacological inhibition of AGEs formation with aminoguanidine significantly reduced viral load both in vitro and in vivo. Mechanistically, AGEs activate p38/ERK/IKK proinflammatory signaling via RAGE and MD2 receptors, leading to epithelial injury and heightened viral susceptibility. Our findings reveal a hyperglycemia-AGEs-RAGE/MD2 axis that increases viral susceptibility in COPD, highlighting AGEs targeting as a promising therapeutic strategy for COPD patients with diabetes.

## Synopsis

Diabetes worsens COPD by promoting airway epithelial accumulation of advanced glycation end products (AGEs), which increase viral susceptibility via RAGE/MD2-dependent pro-inflammatory signaling.

- A higher proportion of frequent acute exacerbations was observed in COPD patients with diabetes and was positively correlated with HbA1c levels.
- Distinct transcriptional alterations in viral infection-related pathways were identified in airway epithelial cells of COPD patients with diabetes by single-cell RNA sequencing.
- AGEs accumulation in the airway epithelium was found to activate p38/ERK/IKK pro-inflammatory signaling through the RAGE and MD2 receptors, leading to epithelial injury and increased viral susceptibility.
- Viral load was significantly reduced by pharmacological inhibition of AGEs both in vitro and in vivo, establishing AGEs as a promising therapeutic target for COPD patients with diabetes.

## Abstract (web summary)

Diabetes worsens COPD by promoting airway epithelial accumulation of advanced glycation end products (AGEs), which increase viral susceptibility via RAGE/MD2‑dependent pro‑inflammatory signaling.

## Graphical abstract

**Figure:**
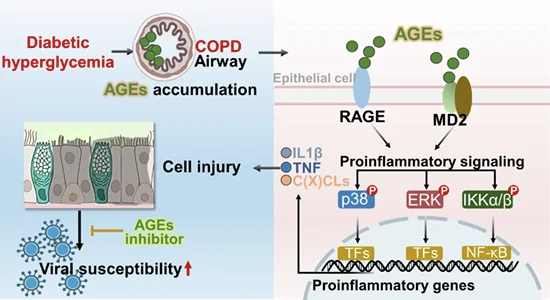

**Figure:**
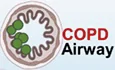

The paper explained **Problem**

Although diabetic complications are well‑established in multiple organs, their impact on the respiratory system, specifically on chronic obstructive pulmonary disease (COPD) progression, remains poorly understood. The mechanisms by which diabetes increases susceptibility to viral infections and exacerbates COPD are unclear.

**Results**

Using single‑cell RNA sequencing of airway epithelium from COPD patients with or without diabetes, we identified distinct transcriptional alterations in host‑defense pathways against viral infection. Functionally, hyperglycemia enhanced influenza virus susceptibility in both COPD‑derived epithelial cells and COPD mice with diabetes. Mechanistically, hyperglycemia promoted accumulation of advanced glycation end products (AGEs) in the airway epithelium, which activated p38/ERK/IKK pro‑inflammatory signaling via RAGE and MD2 receptors, leading to epithelial injury and heightened viral vulnerability. Pharmacological inhibition of AGEs significantly reduced viral load in vitro and in vivo.

**Impact**

Our findings reveal a diabetic hyperglycemia‑AGEs‑RAGE/MD2 axis that drives increased viral susceptibility in COPD, highlighting AGEs as a promising therapeutic target for COPD patients with diabetes.

## Introduction

Chronic obstructive pulmonary disease (COPD) remains a leading global cause of disability and mortality, with airway alteration representing a key pathological hallmark (Christenson et al, 2022). Epidemiological studies indicate that diabetes is one of the most common comorbidities in COPD, affecting ~20% of patients with COPD (Mannino et al, 2015; Cazzola et al, 2017; Fabbri et al, 2023; Anghel et al, 2025). Diabetes is a systemic metabolic disorder characterized by chronic hyperglycemia and causes complications affecting multiple organs, including the retina, kidneys, nerves, and cardiovascular system (Cole and Florez, 2020). While the effects of diabetes on classical target organs are well-characterized, its impact on airway biology remains poorly understood. COPD patients with diabetes face higher risks of adverse outcomes compared to those without diabetes, including elevated risks of frequent COPD acute exacerbations (AECOPD), which is recognized as an important indicator of COPD severity associated with increased risk of hospitalization and mortality (Castan-Abad et al, 2020; Chen et al, 2021; Lin et al, 2021; Foer et al, 2023; Su et al, 2023). These findings indicate pathophysiological interactions between the two conditions, warranting further research to elucidate how diabetes contributes to COPD progression.

The major cause of AECOPD is respiratory infection with viruses, such as influenza A virus (IAV), rhinovirus, and respiratory syncytial virus (RSV), which triggers ~50% of all exacerbation events (Seemungal et al, 2001; Falsey et al, 2006; Beasley et al, 2012; Zwaans et al, 2014; Vogelmeier et al, 2017). The airway epithelial system is the first line of defense against these viruses (Mitlander et al, 2023; Roach et al, 2024; Jarboe et al, 2025). In COPD patients, the airway epithelium exhibits structural and functional defects, including ciliary dysfunction, mucus hypersecretion, and impaired barrier integrity, all of which contribute to increased susceptibility to viral infections (Beasley et al, 2012; Sajjan, 2013; Aghapour et al, 2022). Of note, a study has reported abnormalities in airway epithelial barrier function in diabetic patients (Yu et al, 2024). This suggests that in patients with COPD, a state of diabetic hyperglycemia may increase the risk of AECOPD by impairing the antiviral function of the airway epithelium. Therefore, functional and mechanistic studies are urgently needed to confirm this hypothesis and elucidate the underlying pathogenesis.

Advanced glycation end products (AGEs) is the end products of Maillard reactions between carbonyl compounds and amino compounds. AGEs significantly accumulate during persistent hyperglycemia and play a pivotal role in mediating diabetic complications (Chilelli et al, 2013; Zeng et al, 2019; Wu et al, 2021; Wang et al, 2022; Barutta et al, 2023; Xie et al, 2024). In classical target organs like kidneys, retina, heart, and vasculature, AGEs exert pathogenic effects primarily through the receptor for AGE (RAGE) or myeloid differentiation protein 2 (MD2) binding to promote secretion of pro-inflammatory cytokines and mediate cellular inflammatory damage (Basta et al, 2004; Zong et al, 2011; Chaudhuri et al, 2018; Wang et al, 2020; Wu et al, 2021). However, key unresolved questions persist concerning the airway epithelium in COPD: whether hyperglycemia directly promotes AGEs deposition; whether accumulated AGEs induce structural abnormalities or compromise antiviral defense mechanisms; and ultimately, whether pharmacological inhibition of AGEs could restore epithelial antiviral capacity.

To address these knowledge gaps, we integrated clinical investigations and single-cell RNA sequencing analysis of COPD patients with and without diabetes, in vitro air-liquid interface (ALI) culture model of primary airway epithelial cells derived from COPD patients, and in vivo validation using an established porcine pancreatic elastase (PPE)-induced COPD-like emphysema model combined with streptozotocin (STZ)-induced diabetic hyperglycemia in mice. This study elucidates the mechanistic role of AGEs in diabetic hyperglycemia-driven viral susceptibility of the airway epithelium in COPD, thereby providing a theoretical basis for establishing airway AGEs as therapeutic targets in COPD patients with diabetes.

## Results

### COPD patients with diabetes exhibit increased prevalence of frequent acute exacerbation (AE)

**Figure EV1:**
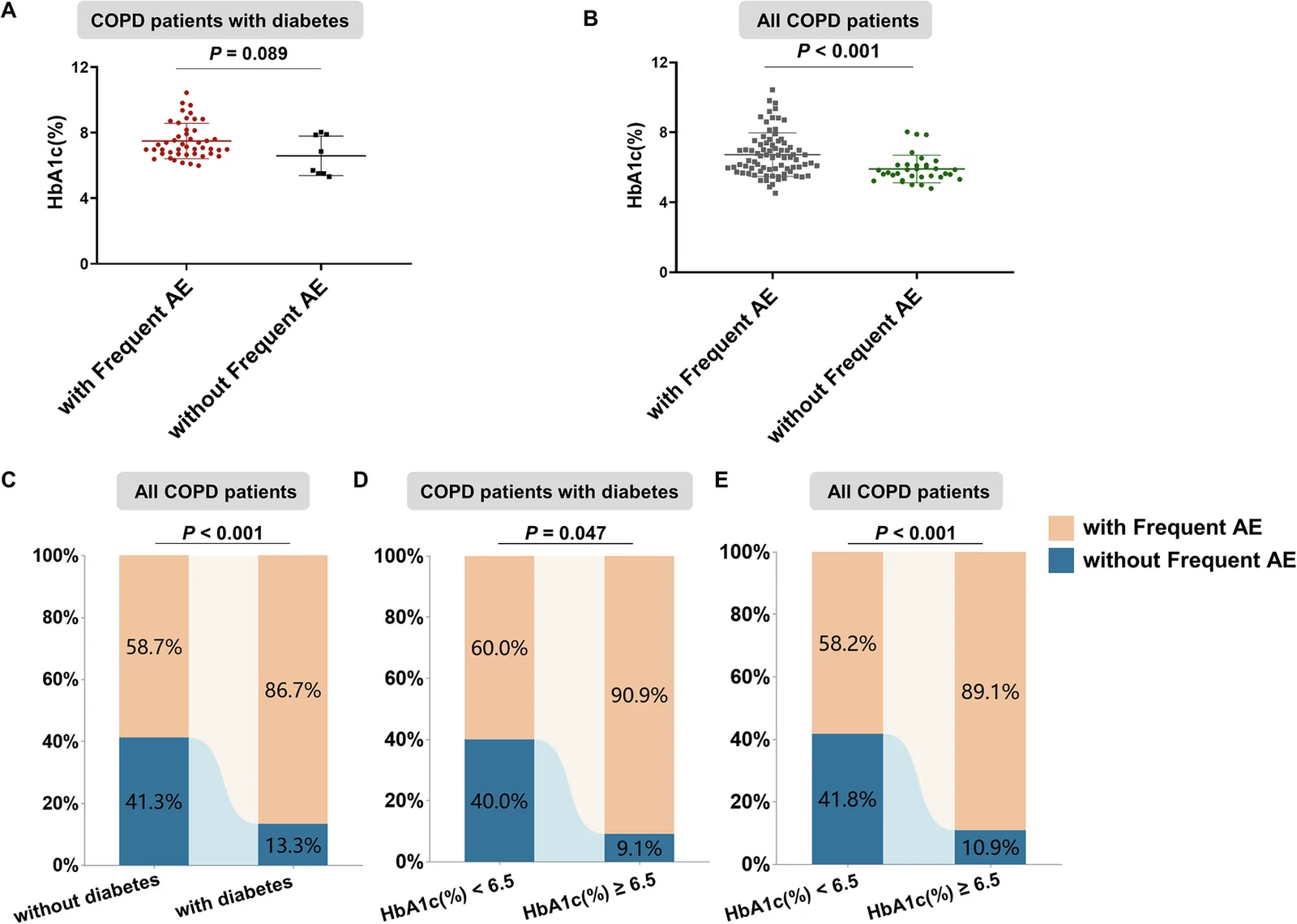
HbA1c level correlates with frequent acute exacerbation (AE) in COPD Cohorts. (**A**,** B**) HbA1c (%) level in the COPD patients with diabetes subgroup (*n* = 60) (**A**) and in the overall COPD cohort (*n* = 123, including non-diabetic subjects) (**B**), stratified by the presence or absence of frequent AE. (**C**) Proportion of patients with or without frequent AE in the overall COPD cohort stratified by those with or without diabetes. (**D**,** E**) Proportion of patients with or without frequent AE in the COPD patients with diabetes subgroup (**D**) and in the overall COPD cohort (**E**), stratified by HbA1c(%) < 6.5 vs. HbA1c(%) ≥ 6.5. Data are shown as Mean ± SD (**A**, B) or as percentage within each group (**C**–**E**). Statistical analyses were performed using the Mann–Whitney test (**A**, **B**), Chi-square with Yates’ correction (**D**) or Chi-square (**C**, **E**). *n* indicates biological replicates.

To investigate the clinical differences between COPD patients with and without diabetes, we conducted a retrospective analysis of clinical data from the First Affiliated Hospital of Guangzhou Medical University. After excluding those with malignancies, asthma, bronchiectasis, or interstitial lung disease, 123 COPD patients were enrolled and then stratified based on diabetes comorbidity status into two groups: COPD patients with diabetes (*n* = 60) and COPD without diabetes (*n* = 63). There are no significant differences in demographic characteristics (gender, age), smoking status (current smoking proportion, pack-year index), disease severity (GOLD stage, pulmonary function parameters), and disease duration between groups (Table EV1). COPD patients are defined as suffering frequent acute exacerbation (AE) if they experienced two or more AE within 1 year. A significantly greater incidence of frequent AE was observed in COPD patients with diabetes compared to their non-diabetes counterparts (86.7 vs 58.7%; *P* = 0.001) (Table EV1). HbA1c is a standard biomarker for diabetes severity monitoring (American, 2025). To analyze the correlation between diabetes and COPD, we further investigated the relationship between HbA1c and frequent AE. As expected, HbA1c levels were higher in COPD patients with diabetes than in those without diabetes (7.05 [6.69–7.95] vs. 5.68 [5.45–6.01]; *P* < 0.001) (Table EV1). Of note, among COPD patients with diabetes, those with frequent AE showed higher HbA1c levels versus those without frequent AE (Fig. EV1A). This correlation between HbA1c and frequent AE was also observed in the overall COPD cohort (Fig. EV1B). In addition, in both populations (COPD patients with diabetes and the overall COPD cohort), we observed that the proportion of individuals with frequent AE was higher in those with diabetes than in those without diabetes (Fig. EV1C), and also higher in those with HbA1c ≥ 6.5% than in those with HbA1c < 6.5%. (Fig. EV1D,E). Taken together, these results confirmed positive associations in both diabetic and overall COPD populations.

### COPD patients with diabetes exhibit compromised defense against viruses in the airway epithelium

**Figure 1:**
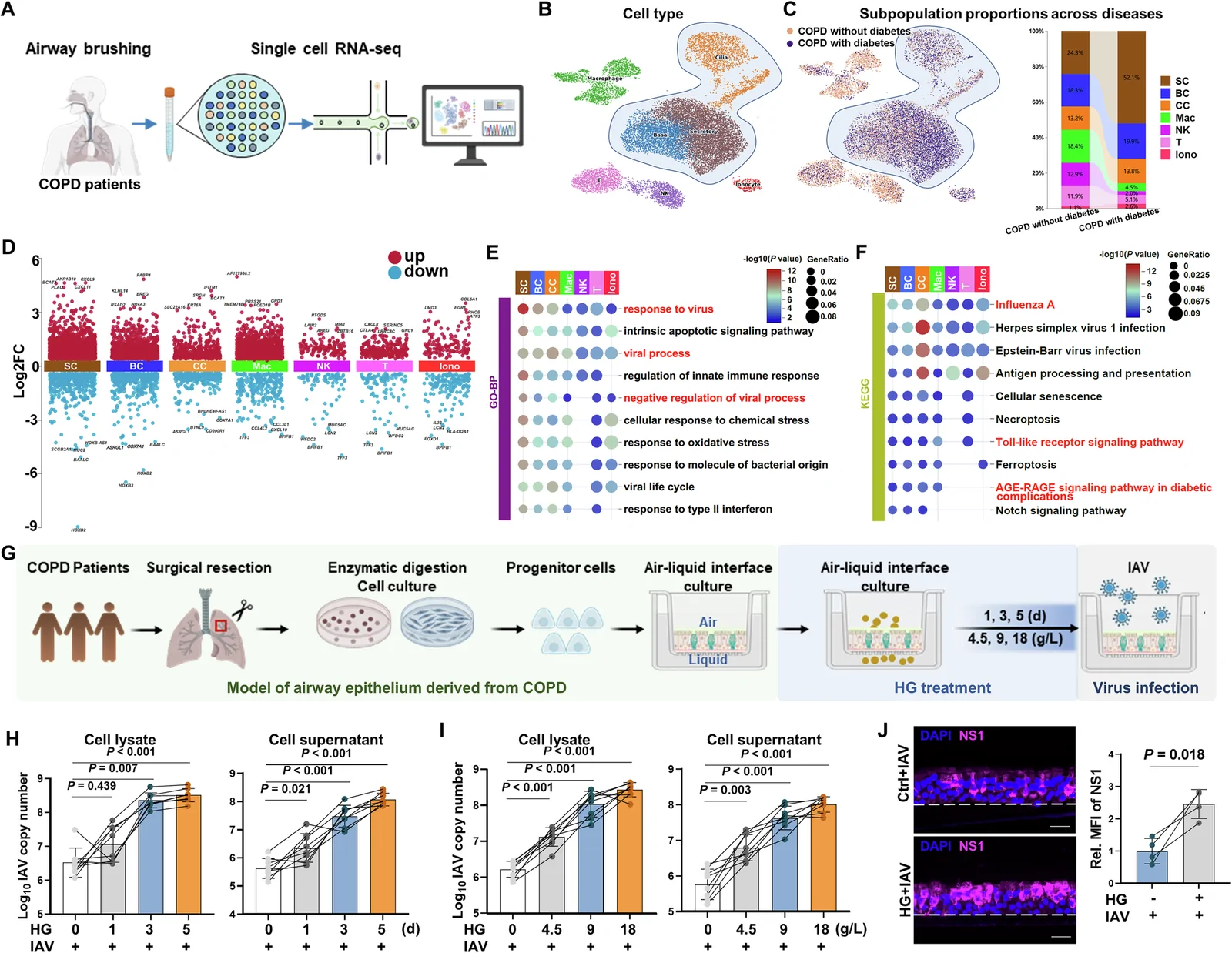
COPD patients with diabetes exhibit compromised defense against viruses in the airway epithelium. (**A**) Experimental design for scRNA-seq analysis; (**B**) Uniform Manifold Approximation and Projection (UMAP) plot showing seven distinct cell clusters. (**C**) Proportional changes in cellular subsets between groups. SC secretory cells, BC basal cells, CC ciliated cells, Mac macrophages, NK NK cells, T T cells, Iono ionocytes. (**D**) Volcano plots of differentially expressed genes (DEGs) in 7 distinct cell clusters. SC, secretory cells; BC, basal cells; CC, ciliated cells; Mac, macrophages; NK, NK cells; T, T cells; Iono, ionocytes. (**E**,** F**) Representative Gene Ontology (GO) (**E**) and Kyoto Encyclopedia of Genes and Genomes (KEGG) (**F**) pathway enrichment analysis of DEGs in seven distinct cell clusters. (**G**) Schematic of ALI-cultured airway epithelium derived from COPD patients treated with HG (0–18 g/L, 0–5 days) followed by IAV infection (MOI = 0.5) and sampling at 24 h post-infection (hpi). (**H**,** I**) Quantification of IAV copy numbers in cell lysate and supernatant from ALI-cultured airway epithelium treated with HG for varying durations (**H**) or concentrations (**I**). *n* = 8. (**J**) Representative immunofluorescence image of IAV nonstructural protein 1 (NS1, purple) in ALI-cultured airway epithelium (side view). Nuclei: DAPI (blue). The dashed line indicates the ALI culture basolateral side. Scale bar: 10 μm. *n* = 4. Data were shown as Mean ± SD and analyzed by the Friedman test with Dunn’s post hoc test (H left panel), RM one-way ANOVA with Dunnett’s post hoc test (H right panel, **I**) or paired *t*-test (**J**). *n* indicates biological replicates. Source data are available online for this figure.

Frequent AE in COPD is closely associated with respiratory viral and bacterial infections, where the airway epithelial barrier serves as one of the primary defenses (Beasley et al, 2012; Aghapour et al, 2022; Mitlander et al, 2023; Roach et al, 2024). To investigate the potential impact of diabetes on the airway epithelium in COPD, we performed single-cell RNA sequencing (scRNA-seq) on bronchial brushing samples obtained from three COPD patients without diabetes and two COPD patients with diabetes (Fig. 1A). The analysis identified seven distinct cell populations based on their canonical marker expression (Fig. 1B; Appendix Fig. S1A). COPD patients with diabetes exhibited altered cellular composition in bronchial brushings, characterized by an increase in epithelial cells (secretory, basal, ciliated cells, and ionocytes) and a decrease in immune cells (macrophages, NK cells, and T cells) (Fig. 1C). Epithelial subpopulation analysis revealed a reduction in ciliated cells and an expansion of secretory cells in the COPD patients with diabetes (Appendix Fig. S1B,C). We further analyzed differentially expressed genes (DEGs) and enrichment signatures across seven cell types. Multi-group volcano plots visualized up/downregulated DEG distributions: secretory cells (2531 up/518 down), basal cells (1369 up/489 down), ciliated cells (462 up/349 down), macrophages (2138 up/255 down), NK cells (141 up/82 down), T cells (261 up/95 down), and ionocytes (136 up/173 down), with top-five up/down-regulated genes annotated (Fig. 1D; Appendix Fig. S1D,E; Table EV2). Enrichment analysis of cell-specific DEGs revealed significant GO terms associated with viral infection responses (Fig. 1E). These responses were more pronounced in airway epithelial cells compared to immune cells, as indicated by lower *P* values (Fig. 1E). These findings suggested that the diabetic comorbidity exerts a stronger influence on viral infection responses in airway epithelial cells than in immune cells in the context of COPD. KEGG analysis demonstrated significant enrichment of multiple viral infection pathways, including influenza A, suggesting altered antiviral defense capacity in COPD patients with diabetes compared with those without diabetes (Fig. 1F). Additionally, observed enrichment of AGE-RAGE and Toll-like receptor pathways highlights key targets for subsequent mechanistic investigation (Fig. 1F).

**Figure EV2:**
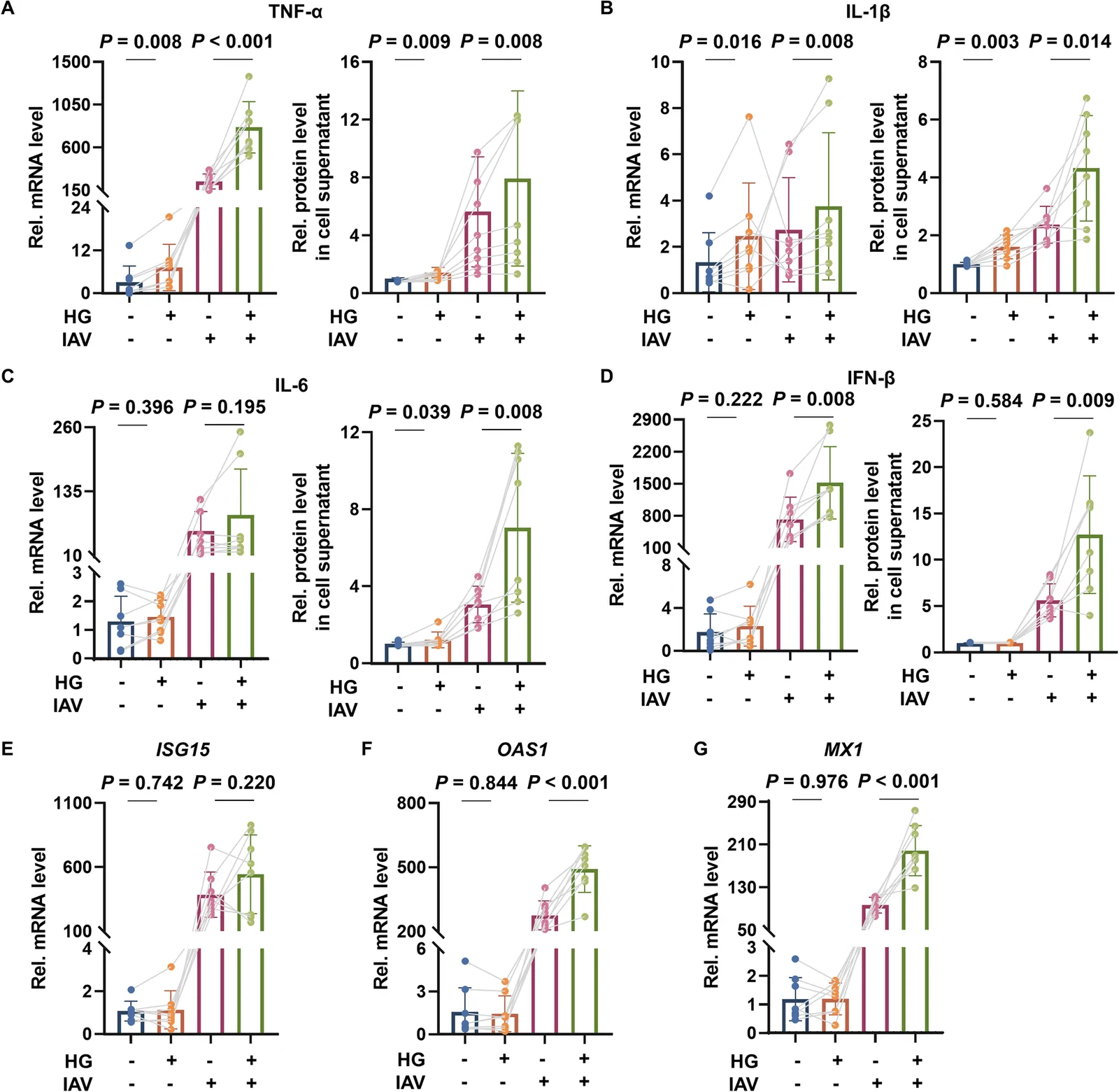
High glucose (HG) potentiates IAV-mediated pro-inflammatory responses in airway epithelium derived from COPD. qPCR (**A**–**D**, left panel;** E**–**G**) and ELISA (**A**–**D**, right panel) measurement of several cytokines and antiviral-related genes in ALI culture treated with or without 9 g/L HG for 3 days, followed by IAV infection (MOI = 0.5, 24 h) or mock infection. *n* = 8. Data were shown as Mean ± SD. The Wilcoxon signed-rank test was used for the following panels: **A** left panel (first two groups), A right panel (last two groups), **B** left panel, C left panel (last two groups), **C** right panel, **D** left panel (last two groups), **E** left panel (first two groups), and **F** left panel (first two groups). The paired *t*-test was used for all other comparisons. *n* indicates biological replicates. Related to Fig. 1.

Diabetes is pathologically characterized by chronic hyperglycemia. Building on enrichment analysis results, we investigated whether hyperglycemia contributes to compromised antiviral function in the airway epithelium of COPD patients. We established ALI-cultured airway epithelium derived from COPD patients and applied high-glucose (HG) stimulation at graded durations (1–5 days) and concentrations (0–18 g/L), followed by IAV infection (Fig. 1G). Quantitative analysis demonstrated that HG pretreatment resulted in dose- and time-dependent increase in viral loads post-infection (Fig. 1H,I), with immunofluorescence imaging confirming increased viral loads in HG-exposed epithelium (Fig. 1J). In parallel, we observed that HG treatment upregulated pro-inflammatory cytokines (TNF-α, IL-1β, and IL-6) (Fig. EV2A–C) but did not alter the expression of interferon‑stimulated genes (ISGs, e.g., IFNB, ISG15, OAS1, and MX1) (Fig. EV2D–G). Following IAV infection after HG treatment, both pro-inflammatory cytokines and ISGs were elevated compared with the IAV-alone group (Fig. EV2A–G). This suggested that the increase in pro-inflammatory cytokines likely results from the combined effects of HG and the virus. In contrast, the upregulation of ISGs is primarily driven by the virus. Notably, the elevated expression of these genes probably reflected a compensatory host response secondary to increased viral loads. Collectively, these results demonstrated that diabetic hyperglycemia potentiates viral susceptibility in the airway epithelium of COPD patients. The underlying mechanisms need further investigation.

### AGEs induce epithelial damage and viral susceptibility in COPD airway epithelium

**Figure 2:**
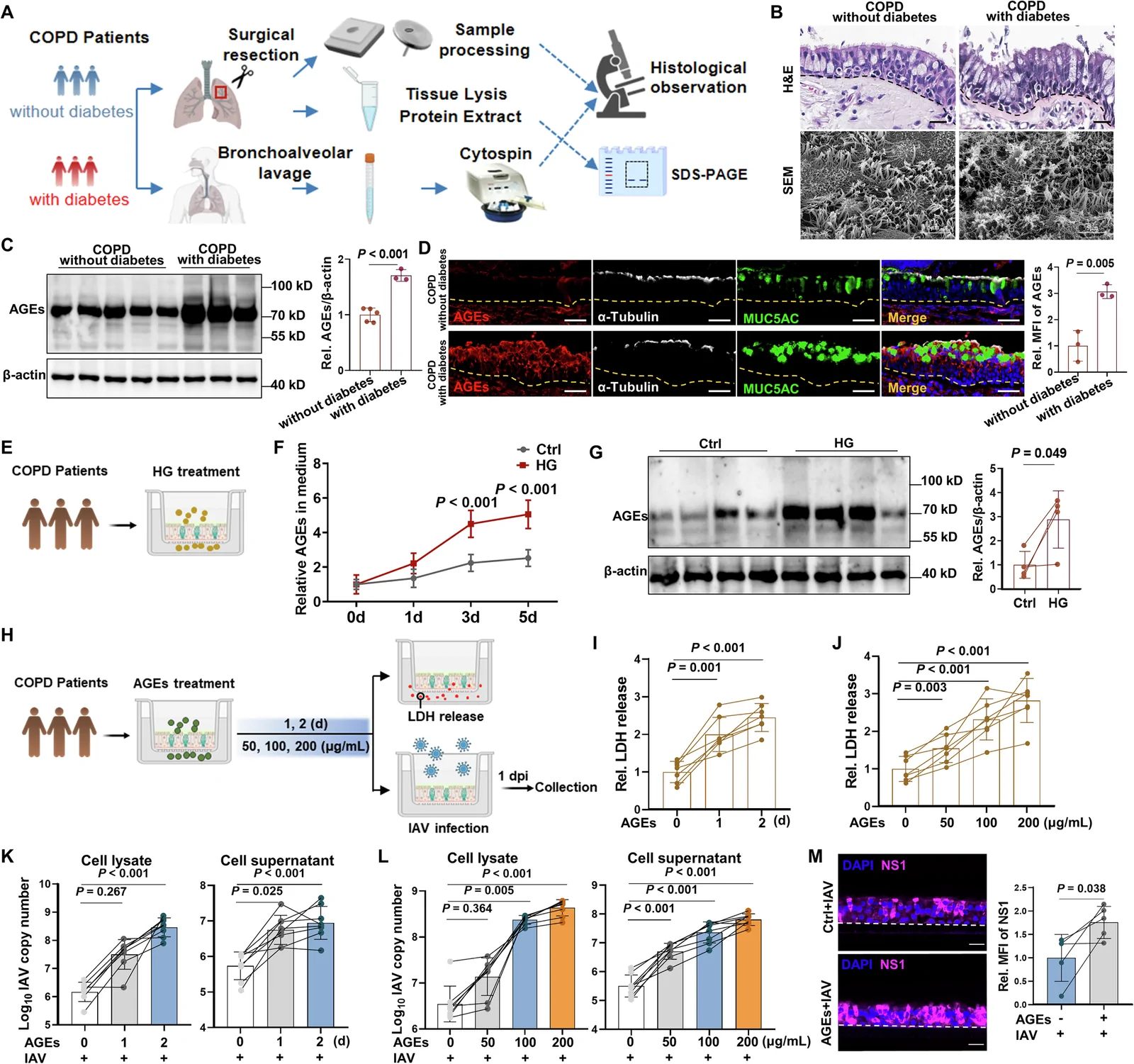
AGEs induce epithelial damage and viral susceptibility in COPD airway epithelium. (**A**) Workflow for the detection of airway epithelial pathological characteristics and AGEs level in bronchial biopsies from COPD with or without diabetes. (**B**) H&E staining and SEM image of bronchial biopsies from COPD with or without diabetes. The dashed line indicates the basal side of the airway mucosal epithelium. Representative images from three independent experiments are shown. Scale bar: 20 μm, 5 μm. (**C**) Quantification of AGEs level in the lysate of bronchial biopsies by WB. *n* = 3–5. (**D**) Representative immunofluorescence image staining for AGEs (red), cilia (α-Tubulin, white) and mucus (MUC5AC, green) in the airways of COPD patients with diabetes. The dashed line indicates the basal side of the airway mucosal epithelium. Scale bar: 50 μm. (**E**) Schematic of ALI-cultured airway epithelium of a COPD patient treated with HG. (**F**) ELISA measurement of AGEs in ALI-cultured airway epithelium supernatant after HG treatment (9 g/L, 0–5 d). *n* = 4. (**G**) Western blot measurement of AGEs in ALI-cultured airway epithelium lysate after HG treatment (9 g/L, 3 d). *n* = 4. (**H**) Experimental design of ALI-cultured airway epithelium exposed to AGEs (0–200 μg/mL, 0–2 days) followed by IAV infection (MOI = 0.5) and sampling at 1 dpi. (**I**) LDH release level in ALI-cultured airway epithelium exposed to 200 μg/mL AGEs for 0–2 days. *n* = 7. (**J**) LDH release level in ALI-cultured airway epithelium treated with different concentrations of AGEs for 1 day. *n* = 7. (**K**,** L**) Quantification of IAV copy numbers in cell lysate and supernatant from ALI-cultured airway epithelium treated with AGEs for varying durations (**K**) or concentrations (**L**). *n* = 8. (**M**) Representative immunofluorescence image of IAV NS1 (purple) in ALI-cultured airway epithelium (side view). Nuclei: DAPI (blue). The dashed line indicates the ALI culture basolateral side. Scale bar: 10 μm. *n* = 5. Data were shown as Mean ± SD and analyzed by unpaired *t*-test (**C**, **D**), RM two-way ANOVA with Bonferroni’s post hoc test (**F**), paired *t-*test (**G**, **M**), RM one-way ANOVA with Dunnett’s post hoc test (**I**, **J**, **L** right panel) and Friedman with Dunn’s post hoc test (**K**, **L** left panel). *n* indicates biological replicates. Source data are available online for this figure.

**Figure EV3:**
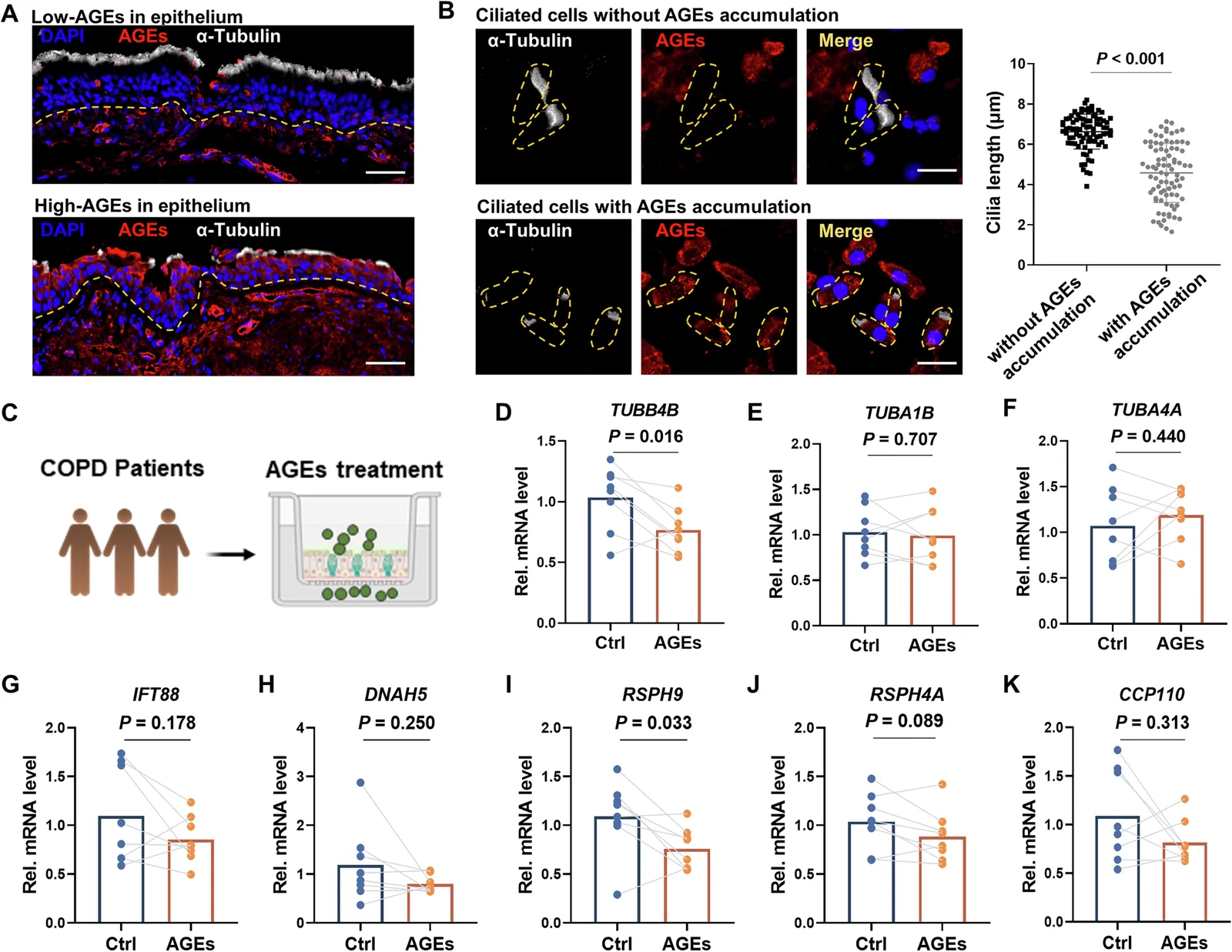
AGEs exposure is associated with epithelial damage. (**A**) Immunofluorescence staining image of airway cilia (α-Tubulin, white) in low-AGEs regions (upper panel, red) or high-AGEs areas (lower panel, red). Nuclei: DAPI (blue). The dashed line indicates the basal side of the airway mucosal epithelium. Representative images from three independent experiments are shown. Scale bar: 50 μm. (**B**) Representative immunofluorescence staining image of cilia (α-Tubulin, white) in BALF-cytospin cells without or with AGEs (red) accumulation. Yellow dashed borders outline individual ciliated epithelial cells. Nuclei: DAPI (blue). Scale bar: 10 μm. *n* = 83–84. (**C**) Schematic of exogenous AGEs exposure in ALI culture. (**D**–**K**) qPCR measurement of several cilia-related genes in ALI culture treated with or without 200 μg/mL AGEs for 1 day. *n* = 8. Data were shown as Mean ± SD and analyzed by unpaired *t*-test (**B**), paired *t*-test (**D**–**G**, **I**, **J**) or Wilcoxon signed-rank test (**H**, **K**). *n* indicates the number of cells (**B**) or biological replicates (**D**–**K**). Related to Fig. 2.

Next, we evaluate pathological characteristics in the airway epithelium of COPD patients with diabetes. Bronchial biopsy specimens were collected from COPD patients with or without diabetes for histopathological analysis (Fig. 2A). The results showed more severe epithelial damage in COPD patients with diabetes, characterized by structurally disorganized cilia (Fig. 2B). As evidenced by the scRNA-seq analysis, the AGE-RAGE signaling, a diabetes-related pathway, was markedly enriched across all three epithelial subtypes (Fig. 1F). Advanced glycation end products (AGEs), the key ligand in this pathway, were initially examined in airway biopsies and bronchoalveolar lavage fluid (BALF) from COPD patients with or without diabetes by using Western blotting and histological staining assays (Fig. 2A). The results showed that COPD patients with diabetes exhibited significantly increased AGEs accumulation in airway tissues (Fig. 2C,D). Detailed histopathological assessment revealed preserved ciliary integrity in low-AGEs regions, whereas high-AGEs areas displayed characteristic ciliary abnormalities, including reduced density and shortened length (Fig. EV3A,B). These results suggested a direct correlation between AGEs accumulation and epithelial damage. In the HG-treated ALI-cultured airway epithelial cells derived from COPD patients (Fig. 2E), we observed a marked elevation of AGEs in both supernatant and cellular lysate (Fig. 2F,G), consistent with findings in airway biopsy tissues from patients and indicating that high glucose promotes AGEs production.

Further, to delineate the mediating role of AGEs in epithelial damage and viral infection, we exposed ALI-cultured airway epithelium derived from COPD patients to exogenous AGEs at varying concentrations and durations, followed by epithelial cytotoxicity measurement or IAV challenge (Fig. 2H). The results revealed that exogenous AGEs exposure induced epithelial cytotoxicity, as evidenced by time- and concentration-dependent LDH release (Fig. 2I,J) and several downregulated cilia-related functional genes (Fig. EV3C–K). Quantitative PCR analysis revealed dose- and time-dependent increases in viral loads following AGEs exposure (Fig. 2K,L), which was further confirmed by immunofluorescence detection of elevated viral loads (Fig. 2M). Meanwhile, AGEs-treated groups exhibited increased IAV-induced inflammatory cytokine production (Appendix Fig. S2). Moreover, immunofluorescence analysis revealed spatial co-localization between AGEs and IAV nucleoprotein (NP), with their expression levels demonstrating a positive correlation trend (Appendix Fig. S3). Collectively, these findings established that overproduction of AGEs directly contributes to airway epithelial damage and viral susceptibility in COPD.

### Pharmacological inhibition of AGEs production attenuates diabetic hyperglycemia-induced viral infection in COPD

**Figure 3:**
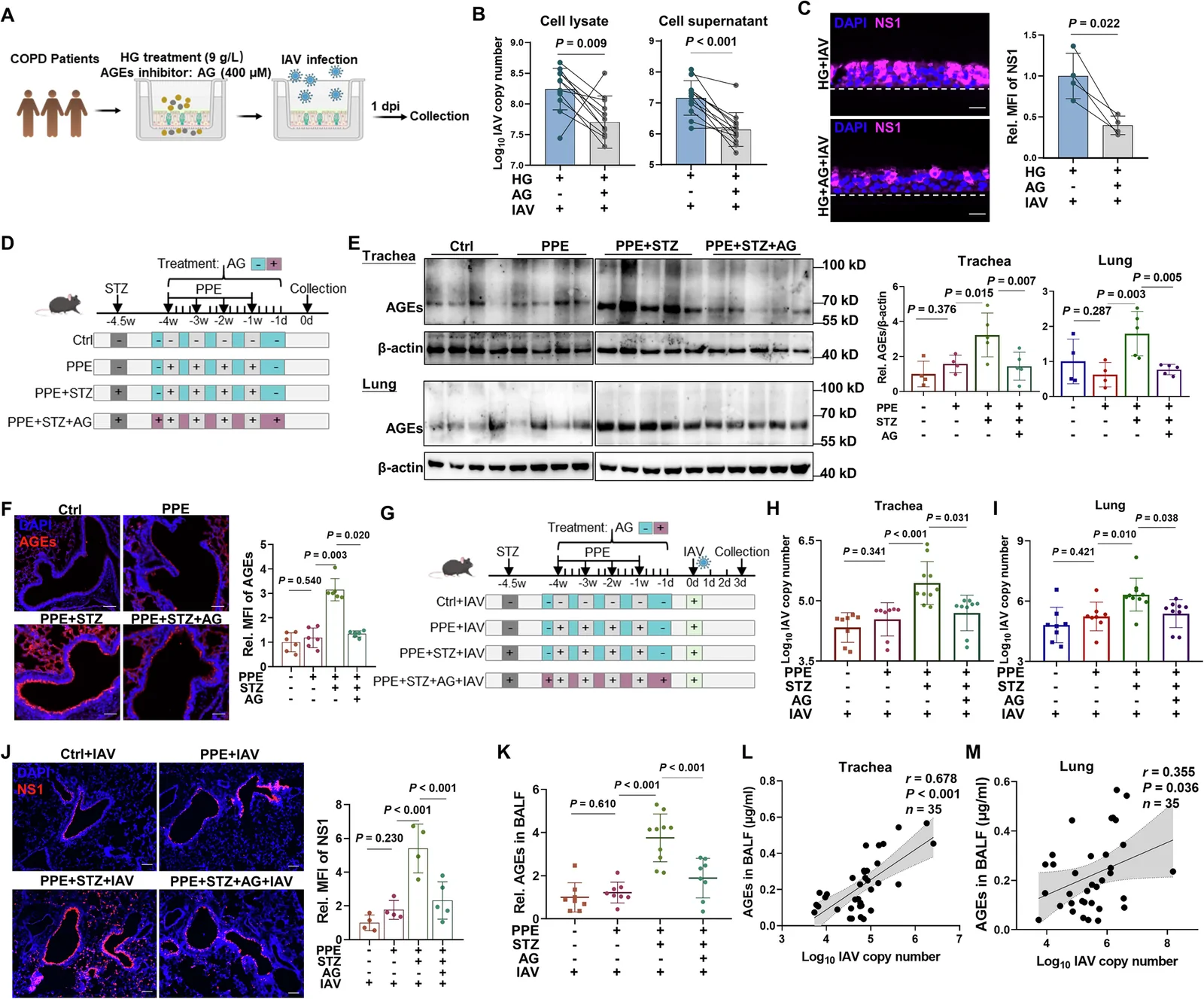
Pharmacological inhibition of AGEs production attenuates diabetic hyperglycemia-induced viral infection in COPD. (**A**) Intervention design showing AGEs inhibitor, aminoguanidine (AG, 400 μM), was added to HG (9 g/L)-treated ALI-cultured airway epithelium, followed by IAV challenge (MOI = 0.5) for 1 day. (**B**) Quantification of IAV copy numbers in cell lysate and supernatant from ALI-cultured airway epithelium treated with HG, accompanied by AG. *n* = 12. (**C**) Representative immunofluorescence image of IAV NS1 (purple) in ALI-cultured airway epithelium (side view). Nuclei: DAPI (blue). The dashed line indicates the ALI culture basolateral side. Scale bar: 10 μm. *n* = 4. (**D**) Schematic showing C57BL/6 mice received STZ (intraperitoneally, 200 mg/kg) and PPE (intranasally, 1.2 U per mouse) to establish a COPD-diabetes model with or without AG treatment (80 mg/kg/day in drinking water). (**E**) Western blot quantification of AGEs level in trachea (upper panel) and lung (lower panel) of COPD-diabetes mice model treated with AG. *n* = 4–5. (**F**) Representative Immunofluorescence image of airway AGEs deposition in the COPD-diabetes mice model treated with AG. Scale bar: 50 μm. *n* = 6. (**G**) Schematic diagram of C57BL/6 mice receiving STZ (intraperitoneally, 200 mg/kg) and PPE (intranasally, 1.2 U per mouse) to establish a COPD-diabetes model with or without AG treatment (80 mg/kg/day in drinking water), followed by IAV infection (intranasally, 5×10^4^ PFU per mouse). (**H**,** I**) Quantification of IAV copy numbers in trachea (**H**) and lung (**I**) of COPD-diabetes mice model. *n* = 8–10. (**J**) Representative immunofluorescence image of IAV NS1 (red) of lung sections in mice. Nuclei: DAPI (blue). Scale bar: 50 μm. *n* = 4–5. (**K**) BALF AGEs levels by ELISA in COPD-diabetes mice model post-IAV infection. *n* = 8–10. (**L**,** M**) Correlation analysis between BALF AGEs and IAV copy number in trachea (**L**) or lung (**M**). Data were shown as Mean ± SD and analyzed by paired *t*-test (**B**, **C**), one-way ANOVA with Fisher’s LSD post hoc test (**E**, **J**, **K**), Kruskal–Wallis with Dunn’s post hoc test (**F**, **H**, **I**) or Spearman’s test (**L**, **M**). *n* indicates biological replicates. Source data are available online for this figure.

We next investigated whether suppressing AGEs generation could inhibit their effect on viral susceptibility by using the AGEs inhibitor aminoguanidine (AG) (Sell et al, 2001; Thornalley, 2003) in hyperglycemic ALI-cultured airway epithelium and mouse models. In the ALI culture model, after confirming the biocompatibility and AGEs-suppressing activity of AG (Appendix Fig. S4), we administered AG to HG-treated airway epithelium prior to IAV challenge (Fig. 3A). The results displayed a significantly decreased viral load in AG-treated airway epithelium (Fig. 3B,C), indicating that AGEs inhibition attenuates hyperglycemia-induced viral infection in vitro in ALI-cultured COPD airway epithelium.

**Figure EV4:**
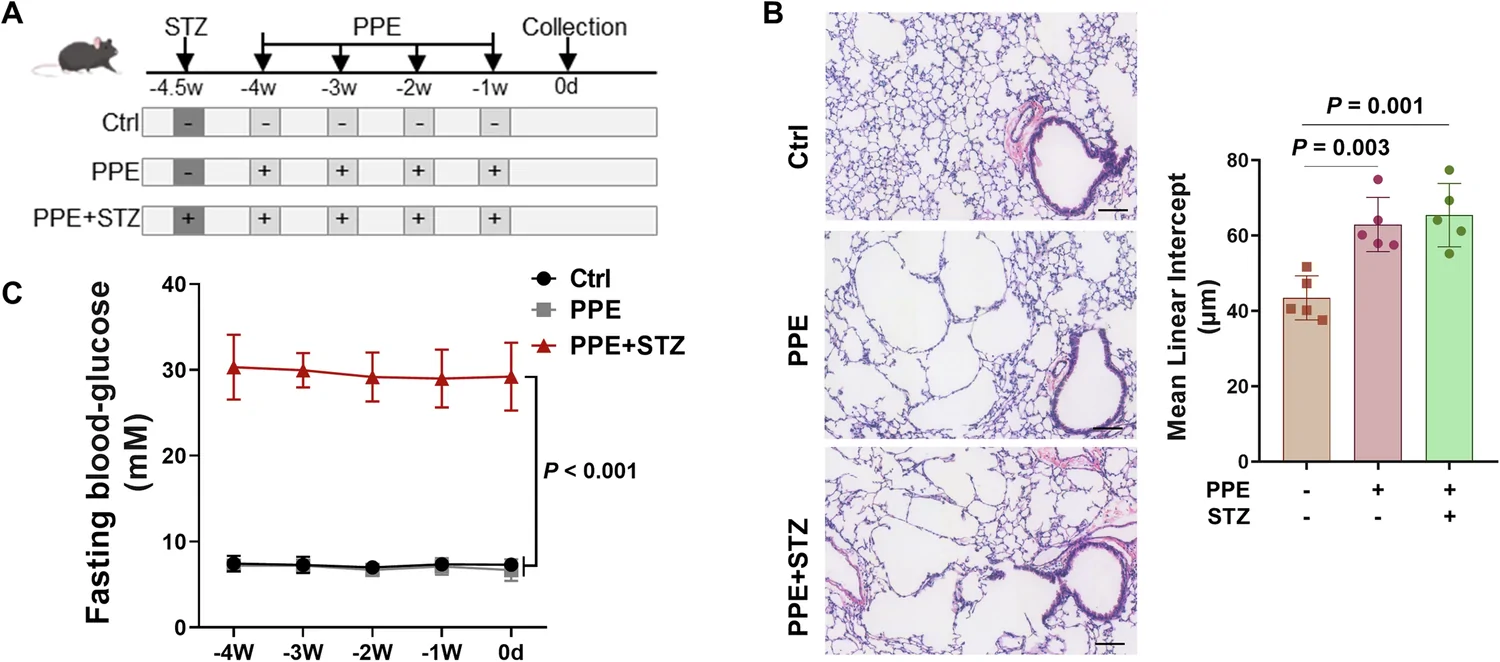
Characterization of COPD-diabetes comorbid mouse model. (**A**) Schematic showing C57BL/6 mice received PPE (porcine pancreatic elastase, intranasal, 1.2 U) + STZ (streptozotocin, intraperitoneal, 200 mg/kg) to establish a COPD-diabetes model. (**B**) Representative images of H&E-stained lung sections and the mean linear intercept (MLI) measurement. Scale bar: 100 μm. *n* = 5. (**C**) Fasting blood glucose levels of the COPD-diabetes mouse model. *n* = 5. Data were shown as Mean ± SD and analyzed by one-way ANOVA with Fisher’s LSD post hoc test. *n* indicates biological replicates. Related to Fig. 3.

To further explore this effect of AGEs inhibition in vivo, we established a mouse model with comorbid COPD and diabetic hyperglycemia via intranasal PPE administration (for COPD-like emphysema induction), combined with intraperitoneal STZ injection (for diabetic hyperglycemia modeling) (Fig. EV4A). Pathological evaluation confirmed successful emphysematous changes (Fig. EV4B), accompanied by significantly elevated fasting blood glucose levels (Fig. EV4C). Following validation of this COPD-diabetic comorbidity model, the AGEs inhibitor aminoguanidine (AG) was administered to experimental mice (Fig. 3D). Western blot and immunofluorescence analyses showed elevated AGEs levels in trachea/lung tissues in PPE + STZ-treated mice, confirming that diabetic hyperglycemia enhances AGEs accumulation in COPD-emphysema models (Fig. 3E,F). Conversely, AG treatment effectively reduced AGEs levels in trachea/lung tissues of COPD-diabetic mice compared with untreated comorbidity models (Fig. 3E,F). The mice were subsequently challenged with IAV (Fig. 3G). Viral loads were significantly elevated in the PPE + STZ group compared with PPE-only controls, while AG treatment effectively reduced viral loads in the PPE + STZ + AG group (Fig. 3H–J). These results indicated that targeted AGEs production alleviates hyperglycemia-driven viral susceptibility in COPD, establishing AGEs as central mediators of infection exacerbation in COPD with diabetes. Furthermore, quantitative analysis of BALF AGEs levels post-viral infection revealed a significant positive correlation with airway/lung viral loads (Fig. 3K–M), suggesting that BALF AGEs may serve as a potential biomarker for respiratory viral loads. Together, these findings conclusively demonstrated that diabetic hyperglycemia induces viral infection in COPD through AGEs-dependent mechanisms, and pharmacological inhibition of AGEs production effectively mitigates this pathological process.

### AGEs bound RAGE/MD2 receptors, activated p38/ERK/IKK phosphorylation, and upregulated pro-inflammatory cytokines in airway epithelium

**Figure 4:**
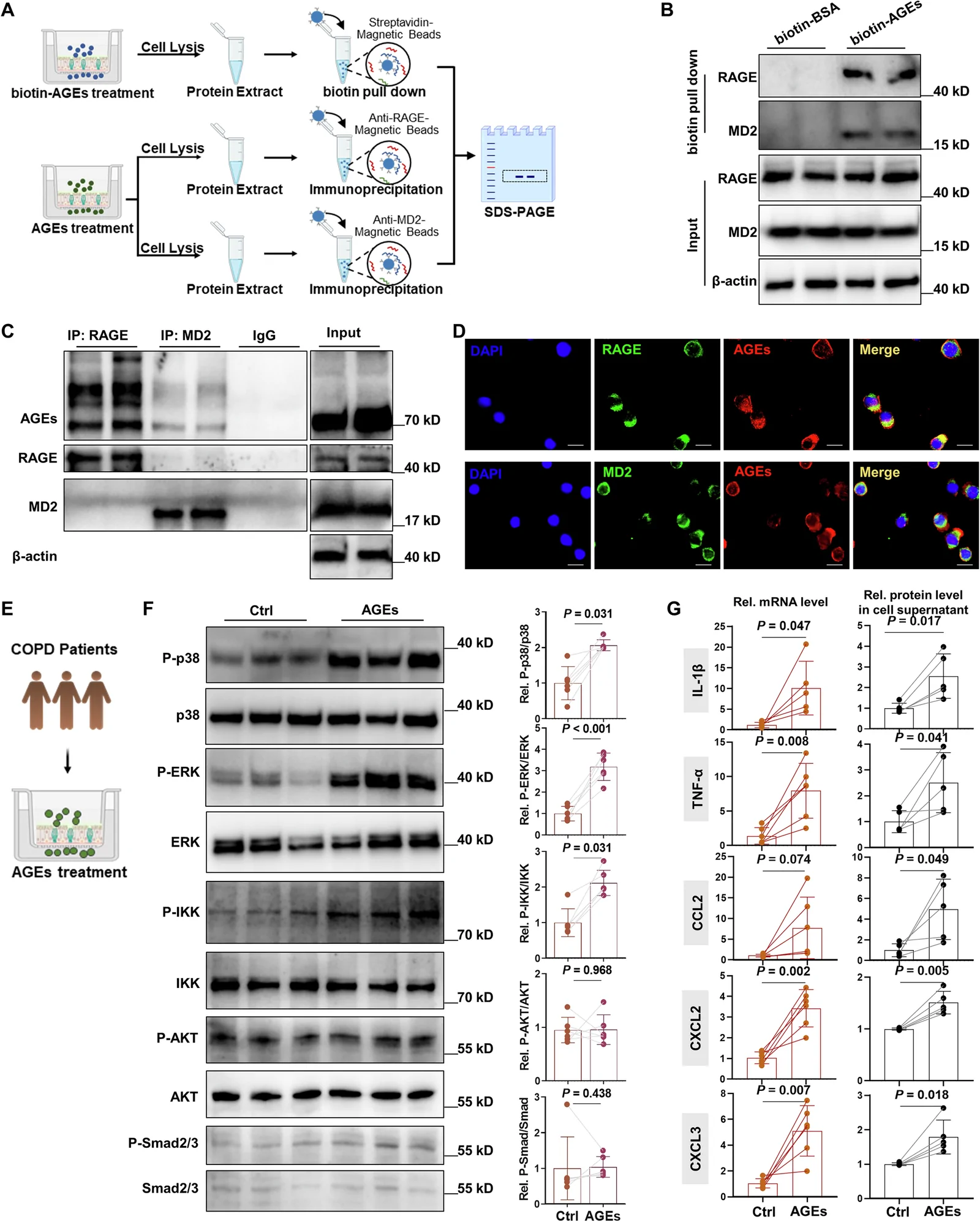
AGEs bound RAGE/MD2 receptors, activated p38/ERK/IKK phosphorylation, and upregulated pro-inflammatory cytokines in airway epithelium. (**A**) Experimental design for biotinylated protein pull-down and co-immunoprecipitation (Co-IP) Assay: (1) biotin-AGEs pull-down for RAGE/MD2 detection; (2) Anti-RAGE Co-IP for AGEs detection; (3) Anti-MD2 Co-IP for AGEs detection. (**B**) Streptavidin bead pull-down products were analyzed by WB using anti-RAGE and anti-MD2 antibodies. (**C**) Immunoprecipitates obtained using anti-RAGE or anti-MD2 antibodies were subjected to western blot analysis, probing for AGEs. (**D**) Immunofluorescence image of subcellular co-localization of AGEs (red) with RAGE (green, upper panel) or MD2 (green, lower panel). Representative images from three independent experiments are shown. Scale bar: 5 μm. (**E**) Schematic of exogenous AGEs exposure in ALI-cultured airway epithelium derived from COPD. (**F**) Western blots quantification of phospho-p38/ERK/IKK/AKT/Smad level in ALI-cultured airway epithelium treated with 200 μg/mL AGEs for 1 h. (**G**) qPCR (left panel) and ELISA (right panel) quantification of several cytokines expression in ALI-cultured airway epithelium (for qPCR) or cell supernatant (for ELISA) after treatment with 200 μg/mL AGEs for 6 h (left panel) or 12 h (right panel). *n* = 5-6. Data were shown as Mean ± SD and analyzed by paired *t*-test (**F** ERK and AKT panel, **G**) or Wilcoxon signed-rank test (**F** p38, IKK and Smad panel). *n* indicates biological replicates. Source data are available online for this figure.

We next investigated the downstream mechanisms by which AGEs induce epithelial cell viral susceptibility in COPD with diabetes. AGEs act through their canonical receptor RAGE (Chaudhuri et al, 2018; Yan et al, 2021) and the recently identified receptor MD2 (Wang et al, 2020) to activate inflammatory pathways and induce the corresponding pathophysiological processes. Of note, the signaling pathways corresponding to these two receptors were both identified in our scRNA-seq analysis (Fig. 1F), thereby implicating them as potential mediators of AGEs-induced airway epithelial abnormalities in COPD. To explore AGE-receptor interactions in airway epithelium, we conducted biotinylated protein pull-down and co-immunoprecipitation (Co-IP) assays in ALI-cultured airway epithelium treated with AGEs (Fig. 4A). The biotinylated protein pull-down assay demonstrated specific capture of RAGE and MD2 proteins by using streptavidin beads to bind biotin-AGEs (Fig. 4B), while Co-IP confirmed efficient precipitation of AGEs by using RAGE/MD2 antibodies (Fig. 4C). Concurrently, immunofluorescence revealed a spatial co-localization of AGEs with either RAGE or MD2 at the subcellular level (Fig. 4D), collectively validating physical interactions between AGEs and both receptors in airway epithelium. Phosphorylation of p38, ERK, and IKK represents a hallmark event in the activation of downstream inflammatory signaling through RAGE/MD2 (Yan et al, 2021; Li et al, 2023). We found that AGEs treatment significantly enhanced the phosphorylation of p38, ERK, and IKK and upregulated inflammatory cytokine expression in COPD-derived ALI-cultured airway epithelium (Fig. 4E–G). In addition, we also examined other downstream signaling pathways of AGEs, including AKT (Yang et al, 2013) and TGFβ/Smad (Fukami et al, 2004), and observed that AGEs did not activate either of these pathways in COPD‑derived ALI‑cultured airway epithelium (Fig. 4F). These results indicated that AGEs potentially activate pro-inflammatory signaling through binding RAGE/MD2 receptors to induce inflammatory cell injury and viral susceptibility in COPD airway epithelium, which requires further experimental validation.

### AGEs potentiate pro-inflammatory signaling, airway epithelial injury and viral susceptibility in COPD with diabetes through RAGE and MD2

**Figure 5:**
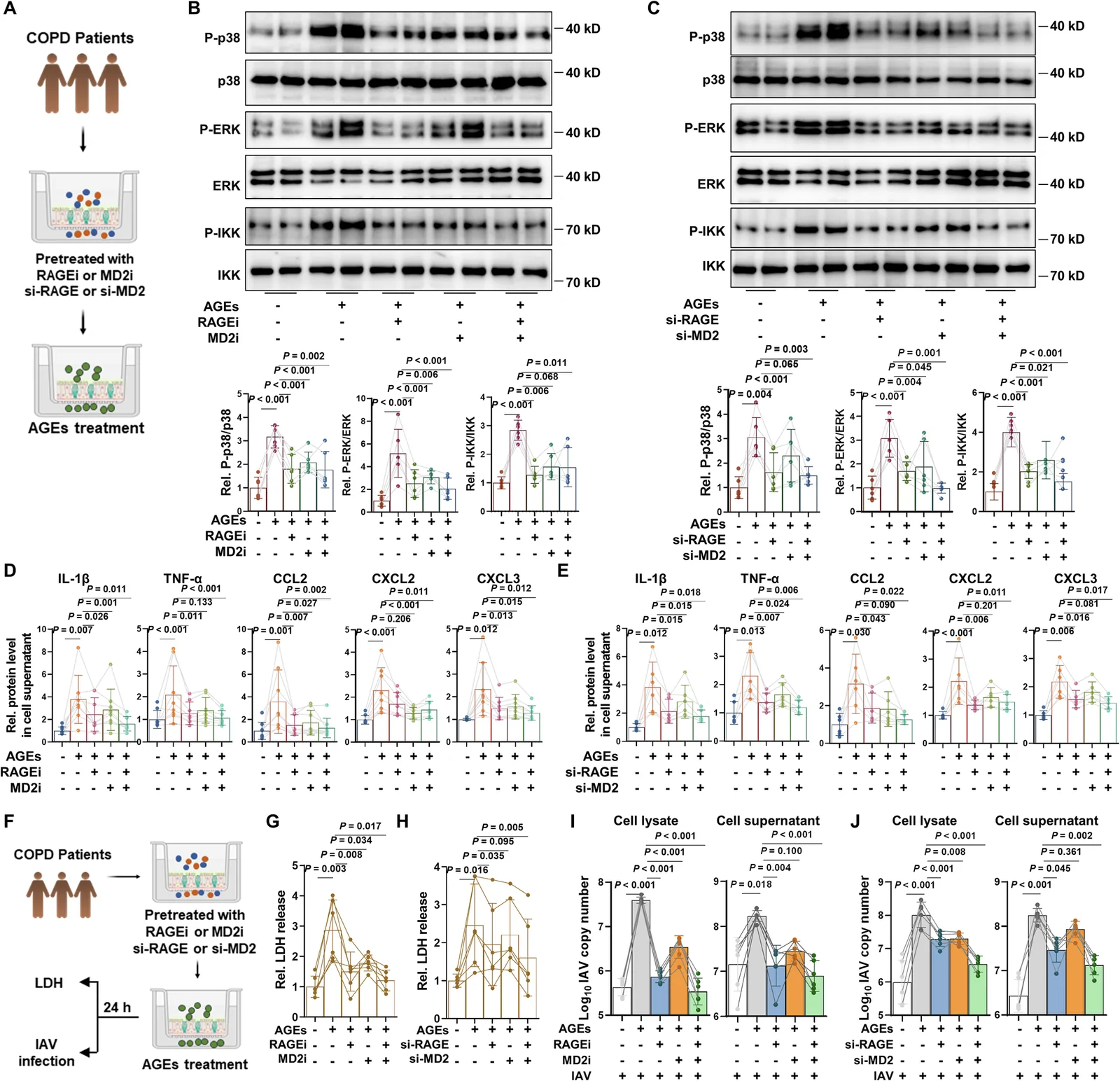
AGEs potentiate pro-inflammatory signaling, airway epithelial injury and viral susceptibility in COPD with diabetes via RAGE and MD2. (**A**) Schematic diagram illustrating the inhibitory effects of pharmacological inhibitors (RAGEi and MD2i) and genetic knockdown (RAGE siRNA and MD2 siRNA) on AGEs‑treated ALI‑cultured airway epithelium derived from COPD patients. (**B**) Western blots quantification of phospho-p38/ERK/IKK in ALI-cultured airway epithelium pretreated with RAGEi (TTP488, 5 μM) and/or MD2i (L6H21, 10 μM) for 2 h followed by 200 μg/mL AGEs treatment for 1 h. *n* = 6. (**C**) Western blot measurement of phospho-p38/ERK/IKK in ALI-cultured airway epithelium transfected with RAGE and/or MD2 siRNA, followed by 200 μg/mL AGEs treatment for 1 h. *n* = 6. (**D**) ELISA measurement of several cytokines expression in ALI-cultured airway epithelium pretreated with RAGEi (TTP488, 5 μM) and/or MD2i (L6H21, 10 μM) for 2 h, followed by 200 μg/mL AGEs for 12 h. *n* = 8. (**E**) ELISA measurement of several cytokines expression in ALI-cultured cell supernatant transfected with si-RAGE and/or si-MD2, followed by 200 μg/mL AGEs for 12 h. *n* = 6. (**F**) Schematic diagram depicting the inhibitory effects of pharmacological inhibitors (RAGEi and MD2i) and genetic knockdown (RAGE siRNA and MD2 siRNA) on AGEs-induced cytotoxicity (LDH release) and IAV infection susceptibility in ALI-cultured airway epithelium. (**G**) LDH release level in ALI-cultured airway epithelium pretreated with RAGEi (TTP488, 5 μM) and/or MD2i (L6H21, 10 μM) for 2 h, followed by 200 μg/mL AGEs for 24 h. *n* = 6. (**H**) LDH release level in ALI-cultured airway epithelium transfected with RAGE and/or MD2 siRNA, followed by 200 μg/mL AGEs for 24 h. *n* = 6. (**I**) Quantification of IAV copy numbers in cell lysate and supernatant from ALI-cultured airway epithelium pretreated with RAGEi (TTP488, 5 μM) and/or MD2i (L6H21, 10 μM) for 2 h, followed by 200 μg/mL AGEs for 24 h and IAV infection (MOI = 0.5) at 1 dpi. *n* = 6. (**J**) Quantification of IAV copy numbers in cell lysate and supernatant from ALI-cultured airway epithelium transfected with RAGE and/or MD2 siRNA, followed by 200 μg/mL AGEs for 24 h and IAV infection (MOI = 0.5) at 1 dpi. *n* = 6. Data were shown as Mean ± SD and analyzed by Friedman with Dunn’s post hoc test (**B** upper panel, **D** middle panel, **E** penultimate panel, **I** right panel, **J** right panel) or RM one-way ANOVA with Fisher’s LSD post hoc test (**B** middle and lower panel, **C**, **D** left and right panel, **E** remaining panel, **G**–**I** left panel, **J** left panel). *n* indicates biological replicates. Source data are available online for this figure.

Subsequently, using pharmacological inhibitors (RAGE inhibitor TTP488 (Burstein et al, 2018)) and (MD2 inhibitor L6H21 (Wang et al, 2015)) or genetic knockdown (RAGE siRNA and MD2 siRNA) prior to AGEs stimulation (Fig. 5A; Appendix Fig. S5), we investigated their respective contributions to AGEs-induced pro-inflammatory signaling activation, airway epithelial damage and viral susceptibility.

Results showed that individual inhibition or knockdown of either RAGE or MD2 partially attenuated AGEs-upregulated p38/ERK/IKK phosphorylation and cytokine expression, while dual receptor inhibition appeared to result in an enhanced suppression (Fig. 5B–E). These results established that AGEs exert pro-inflammatory effects through dual receptor engagement in airway epithelial cells.

**Figure EV5:**
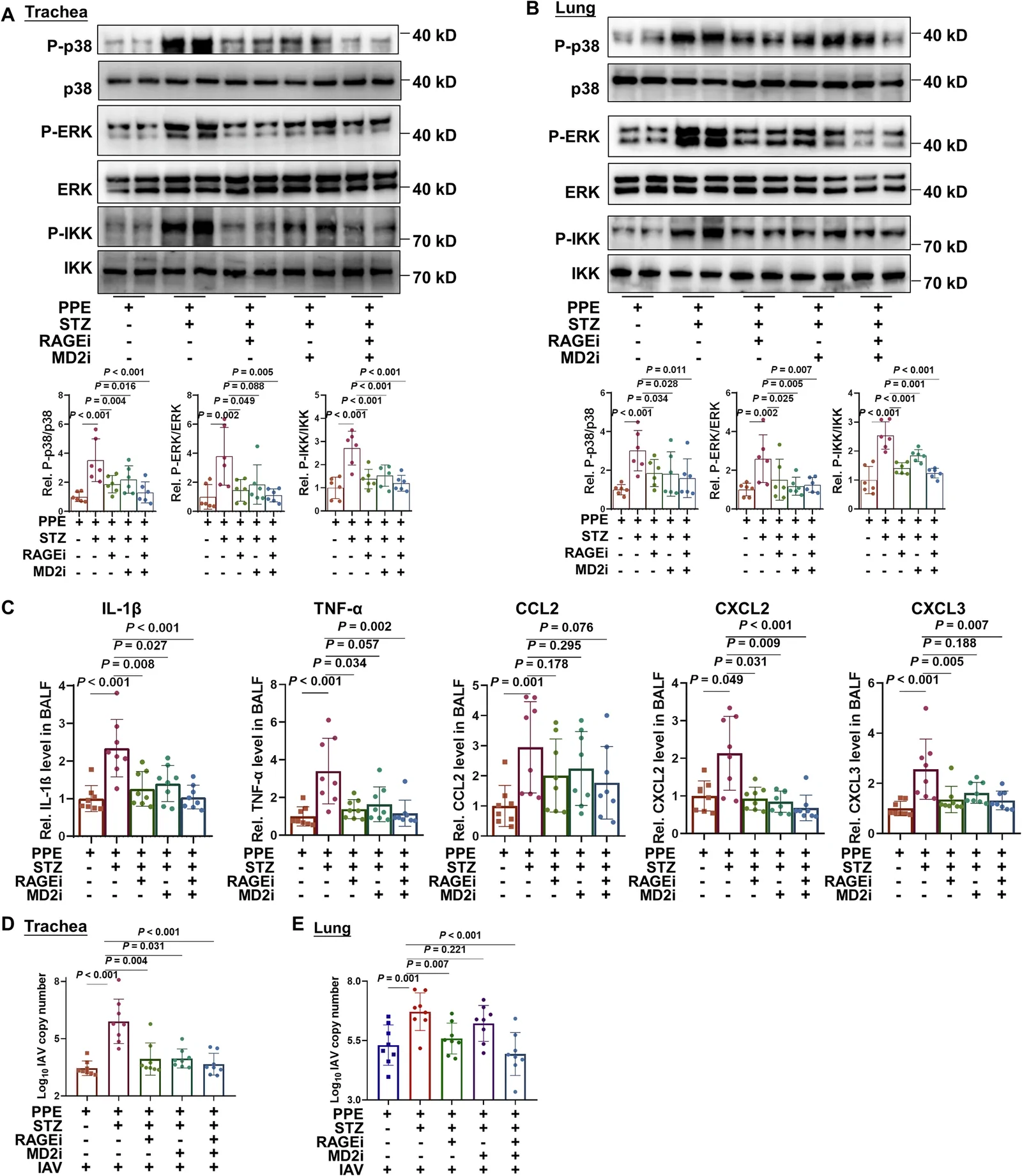
RAGE and MD2 mediate diabetic hyperglycemia-induced pro-inflammatory signaling activation and viral susceptibility in COPD mice with diabetes. (**A**,** B**) Western blot measurement of phospho-p38/ERK/IKK in trachea (**A**) and lung (**B**) of COPD-diabetes mice model treated with RAGEi or MD2i. *n* = 6. (**C**) ELISA measurement of several cytokines expression in BALF of COPD-diabetes mice model treated with RAGEi or MD2i. *n* = 8. (**D**,** E**) Quantification of IAV copy numbers in trachea (**D**) and lung (**E**) of COPD-diabetes mice model. *n* = 8. Data were shown as Mean ± SD and analyzed by one-way ANOVA with Fisher’s LSD post hoc test (**A** left and right panel, **B**, **E**) or Kruskal–Wallis with Dunn’s post hoc test (**A** middle panel, **C**, **D**). *n* indicates biological replicates. Related to Fig. 5.

Furthermore, inhibition or knockdown of RAGE or MD2 also attenuated AGEs-induced airway epithelial injury, validated by decreased LDH release, and viral susceptibility confirmed by decreased viral loads in COPD-derived ALI-cultured airway epithelium (Fig. 5F–J). Notably, the protective effect of RAGE inhibition or knockdown was somewhat greater than that of MD2, and combined inhibition of both receptors provided greater protective effects (Fig. 5F–J). These results suggest that RAGE may play a more dominant role than MD2 in mediating AGE-induced pathology. However, both receptors contribute to the process, and their combined targeting appears to yield additive/synergistic effects. Taken together, these findings indicate that RAGE and MD2 pro-inflammatory signaling may serve as the dual regulatory axis underlying AGEs-induced epithelial dysfunction and enhanced viral vulnerability in COPD. We further validated the roles of RAGE and MD2 in vivo using COPD mice with diabetes, in which AGEs levels were elevated. Consistent with the in vitro findings, treatment with RAGE or MD2 inhibitors significantly reduced viral loads and downregulated pro‑inflammatory signaling activation as well as the expression of antiviral genes and cytokines in COPD mice with diabetes, indicating their potential for in vivo application (Fig. EV5A–E).

## Discussion

Our study demonstrated that in the context of COPD-diabetes comorbidity, hyperglycemia-induced accumulation of AGEs in the airway epithelium activates RAGE and MD2 pro-inflammatory signaling pathways, mediating epithelial cell injury and increasing viral susceptibility. Comorbidity represents an increasingly critical factor in COPD progression, which complicates disease management and treatment (Cavailles et al, 2013). Research indicates that COPD frequently coexists with diabetes, critically impacting disease outcomes (Anghel et al, 2025). Chronic exposure of tissues to diabetic hyperglycemia is a primary pathogenic factor in diabetes. It is reported that increasing blood glucose concentrations are associated with adverse clinical outcomes in AECOPD patients (Baker et al, 2006). Notably, many diabetic patients develop severe micro- and macro-vascular complications despite glycemic control, a phenomenon attributed to hyperglycemia-induced metabolic memory wherein AGEs accumulation serves as a key mediator (Zeng et al, 2019; Taylor et al, 2021; Wu et al, 2021). HbA1c, a routine clinical test for diabetes monitoring, is one of the most studied and clinically utilized AGEs that are formed via a nonenzymatic reaction between glucose and the amino groups (valine/lysine) of hemoglobin (Dehnad et al, 2020). Although some studies have reported associations between AGEs and COPD, current evidences are largely confined to review articles proposing hypothetical links and correlational analyses from clinical observations (Wu et al, 2011; Gopal et al, 2014; Hoonhorst et al, 2014; Hoonhorst et al, 2016; Sharma et al, 2021; Reynaert et al, 2023). No studies have established a causal relationship between AGEs and COPD, and the specific role of diabetic AGEs in mediating COPD progression remains unclear. This study establishes a causal link between AGEs and COPD progression, whereby diabetic hyperglycemia-induced accumulation of AGEs enhances viral susceptibility in COPD and provides critical insights into the clinical observation of poorer outcomes in COPD patients with diabetes.

AGEs have been extensively implicated in diabetic complications. In diabetic cardiomyopathy, AGEs induce cardiomyocyte ferroptosis and initiate cardiac inflammation, remodeling (cardiomyocyte hypertrophy, pro-fibrotic responses, and fibrosis), and eventually, cardiac dysfunction (Wang et al, 2022). In diabetic angiopathy, the accumulation of AGEs enhances vascular calcification through osteogenic transition and apoptosis of vascular smooth muscle cells (Xie et al, 2024). In diabetic osteopathy, AGEs hinder the normal binding of integrin and other matrix proteins to collagen, affecting osteoblast and osteoclast activity (Gao et al, 2024). Extending this paradigm, our work identifies the airway epithelium as a previously unrecognized target organ for AGEs-induced damage. The concept of “diabetic airway disease” gains substantial support from our observations of AGEs-induced epithelial dysfunction and antiviral defense suppression.

Mechanistically, AGEs may exert pathological effects through multiple interconnected pathways. The canonical receptor RAGE induces inflammatory signaling upon AGEs binding, leading to cellular inflammatory damage (Ott et al, 2014; Manigrasso et al, 2021; Yan et al, 2021). Although RAGE expression is low in the airway epithelium (predominantly expressed in type 1 alveolar cells and leukocytes), our study confirmed its functional presence in the COPD airway epithelium, where AGE-RAGE interactions drive epithelial injury and impaired antiviral defense. Beyond RAGE, Wang et al. have identified MD2, a co-receptor essential for Toll-like receptor 4 (TLR4)-induced pro-inflammatory signaling, as a novel AGEs receptor (Wang et al, 2020). They further revealed that AGE-MD2 binding activates TLR4 pathways, promotes pro-inflammatory cytokine secretion, and drives diabetic cardiomyopathy (Wang et al, 2020). Crucially, we established the parallel role of MD2 in mediating AGE-induced airway epithelial damage and antiviral impairment in COPD. Collectively, these findings revealed a cooperative RAGE-MD2 interaction in disease pathophysiology. This aligns with previously reported RAGE-TLR4 crosstalk, which mediates aberrant T-cell immunity by promoting Th2 responses through RAGE-TLR4 interplay in human peripheral immune cells (Zhang et al, 2025). Our work extends this paradigm by confirming RAGE-MD2 axis functionality in the COPD airway epithelium. We further validated that AGEs activate the p38 MAPK, ERK, and IKK pathways via RAGE and MD2. This is consistent with previous reports showing that both receptors can activate these cascades. Mechanistically, Yan et al reported that SLP76 binds the cytosolic tail of RAGE, activating p38, ERK, and IKK (Yan et al, 2021). Wang et al demonstrated that the TLR4/MD2 complex, upon ligand stimulation (e.g., saturated palmitic acid or high glucose), triggers ERK, p38, and IKK/NF-κB signaling (Wang et al, 2017; Wang et al, 2020). Thus, our observation that ERK, p38 MAPK, and IKK are common downstream effectors of both RAGE and MD2 aligns with the literature.

Clinical translation of these findings presents two primary avenues. First, stratification strategies could identify high-risk COPD patients through quantification of airway AGEs levels in sputum, bronchoalveolar lavage fluid, or bronchial biopsies. Elevated AGEs concentrations might predict exacerbation susceptibility and warrant intensified glycemic control or prophylactic antiviral therapies. Second, pharmacological targeting of the AGEs demonstrates therapeutic promise. AGEs inhibitor treatment in murine models achieved a reduction in IAV viral loads. Inhaled delivery of AGEs inhibitors using biodegradable nanoparticles could enhance lung retention while minimizing systemic toxicity compared to oral formulations. Future directions should focus on optimizing inhaled AGEs inhibitors, developing predictive biomarkers for patients, and conducting multi-ethnic clinical trials to validate these concepts.

There are several limitations in our study. First, the employed animal models (STZ-induced diabetes, elastase-induced emphysema) do not fully replicate human disease complexity, particularly T2D insulin resistance and COPD heterogeneous phenotypes. Second, all diabetic patients in our cohort had T2D, with no T1D cases identified; therefore, we did not stratify our analysis by diabetes type. Nevertheless, although the etiologies of T1D and T2D differ, both conditions converge on chronic hyperglycemia, which is the key driver of diabetic complications. Moreover, AGEs, the central molecule of this study and a key downstream product of hyperglycemia, are elevated in both types of diabetes (Thomas et al, 2015; Maran et al, 2022), suggesting that similar findings may occur in both T1D and T2D populations. Third, we lacked a control group of diabetic patients without COPD, because bronchial biopsies are not routinely obtained from asymptomatic diabetic individuals for ethical reasons. Consequently, we could not directly investigate whether the altered antiviral defense observed in diabetic COPD patients is driven by diabetes itself or by the combination of diabetes and COPD. It should be noted, however, that recent reports have established that diabetes is associated with impaired basal antiviral responses, rendering diabetic individuals more susceptible to respiratory viral infections (Nobs et al, 2023; Estan et al, 2024; Gray et al, 2024). These findings suggest that a diabetes‑induced baseline antiviral defect may also be present in the airway epithelium, thereby contributing to heightened viral susceptibility and more severe COPD. Further studies are warranted to explore this possibility directly. Despite these limitations, we believe this work elucidates the pathogenic cascade linking diabetic hyperglycemia-induced AGEs accumulation to epithelial cell injury and antiviral impairment in COPD, and establishes AGEs inhibitors as a novel therapeutic paradigm for breaking the diabetes-COPD comorbidity cycle.

## Methods

**Table:** Reagents and tools table

Reagent/resource	Reference or source	Identifier or catalog number
**Experimental models**		
C57BL/6 J	GemPharmatech	N/A
Human Airway epithelial cell	This study	N/A
**Antibodies**		
Anti-AGEs (WB 1:1000; IF 1: 300)	Abcam	ab23722
Anti-α-Tubulin (acetyl K40) (IF 1: 300)	Abcam	ab289863
Anti-MUC5AC (IF 1: 100)	Thermo Fisher	MA5-12178
Anti-KRT5 (IF 1: 300)	Abcam	ab52635
Anti-IAV NS1 (IF 1: 300)	GeneTex	GTX125990
Anti-IAV NP (IF 1: 300)	GeneTex	GTX629633
Anti-RAGE (WB 1: 500; IF 1: 100)	Santa Cruz	sc-365154
Anti-RAGE (IP 1: 100)	Immunoway	YM8606
Anti-MD2 (IP 1: 100)	Proteintech	11784-1-AP
Anti-MD2 (IF 1: 100)	Santa Cruz	sc-80183
Anti-MD2 (WB 1:1000)	Immunoway	YN2063
Anti-P-p38 (Thr180/Tyr182) (WB 1:1000)	Proteintech	28796-1-AP
Anti-p38 (WB 1:1000)	CST	8690
Anti-P-ERK (Thr202/Tyr204) (WB 1:1000)	CST	4370
Anti-ERK (WB 1:1000)	CST	4695
Anti-P-IKK (Ser176/180) (WB 1:1000)	Immunoway	YM8579
Anti-IKK (WB 1:1000)	Immunoway	YM8275
Anti-P-Smad2 (Ser465/467)/Smad3 (Ser423/425) (WB 1:1000)	Proteintech	80427-2-RR
Anti-Smad2/3 (WB 1:1000)	Immunoway	YT4332
Anti-P-AKT (Ser473) (WB 1:1000)	Immunoway	YM8304
Anti-AKT (WB 1:1000)	Immunoway	YM8643
**Oligonucleotides and other sequence-based reagents**		
siRNA-negative control (NC)	GenePharma	Sense: 5’-UUCUCCGAACGU GUCACGUTT-3’ Antisense: 5’- ACGUGACACG UUCGGAGAATT-3'
siRNA-RAGE	GenePharma	Sense: 5’-GCCUCUGGAGGA GGUCCAATT-3’ Antisense: 5’- UUGGACCUCC UCCAGAGGCTT -3'
siRNA-MD2	GenePharma	Sense: 5’-CUCAUCCGAUGC AAGUAUUTT-3’ Antisense: 5’-AAUACUUGCA UCGGAUGAGTT-3'
qRT-PCR primers	BGI Genomics	Table EV4
**Chemicals, enzymes and other reagents**		
Mannitol	Sigma	PHR1007
D-(+)-Glucose	Sigma	G7021
BSA	Sigma	A1933
AGE-BSA	abcam	ab51995
Biotin-conjugated BSA	FineTest	FNSA-0159
Biotinylated AGE-BSA	R&D	BT4127
Porcine pancreatic elastase (PPE)	Macklin	E808882
Streptozocin (STZ)	Sigma-Aldrich	S0130
Aminoguanidine	Selleck	S4548
Azeliragon (TTP488)	Selleck	S6415
L6H21	MCE	HY-W082785A
Human IL-1β ELISA Kit	ELK Biotechnology	ELK1270
Human TNF-α ELISA Kit	ELK Biotechnology	ELK1190
Human CCL2 ELISA Kit	ELK Biotechnology	ELK5252
Human CXCL2 ELISA Kit	ELK Biotechnology	ELK2079
Human IL-6 ELISA Kit	ELK Biotechnology	ELK1156
Human IFN-β ELISA Kit	ELK Biotechnology	ELK1101
Human CXCL3 ELISA Kit	ELK Biotechnology	ELK10361
Mouse IL-1β ELISA Kit	ELK Biotechnology	ELK1271
Mouse TNF-α ELISA Kit	ELK Biotechnology	ELK1387
Mouse CCL2 ELISA Kit	ELK Biotechnology	ELK5056
Mouse CXCL2 ELISA Kit	ELK Biotechnology	ELK1383
Mouse CXCL3 ELISA Kit	ELK Biotechnology	ELK0457
AGEs ELISA kit	mlbio	ml024019
**Software**		
ImageJ	National Institutes of Health	ImageJ 1.51k
GraphPad Prism 9	GraphPad	Prism 8.0.1

### Clinical data collection

Clinical data were retrospectively collected from patients at The First Affiliated Hospital of Guangzhou Medical University between January 2019 and December 2024. COPD was diagnosed according to the Global Initiative for Chronic Obstructive Lung Disease (GOLD) guidelines (Singh et al, 2019). Patients presenting with relevant symptoms (e.g., dyspnea, chronic cough, and sputum production) and a history of risk factor exposure (e.g., cigarette smoke and environmental pollutants) were considered for the diagnosis, which was confirmed by a post‑bronchodilator FEV1/FVC ratio of less than 0.70. Diabetes was diagnosed based on the American Diabetes Association (ADA) Standards of Medical Care in Diabetes, meeting any of the following four criteria: A1C ≥6.5%, fasting plasma glucose ≥126 mg/dL, 2‑hour plasma glucose during OGTT ≥200 mg/dL, or random plasma glucose ≥200 mg/dL accompanied by classic hyperglycemic symptoms (American, 2019). Following exclusion of individuals with malignancies, asthma, bronchiectasis, or interstitial lung disease, 123 participants were enrolled, comprising 63 COPD patients without diabetes and 60 COPD patients with diabetes. All diabetic patients included in this cohort had type 2 diabetes (T2D).

### Ethics for human sample collection

Human lung tissues, bronchial brushings, and bronchoalveolar lavage fluid (BALF) from COPD patients with or without diabetes were obtained from The First Affiliated Hospital of Guangzhou Medical University. All diabetic patients included in this cohort had type 2 diabetes (T2D). Informed consent was obtained from all subjects. The study protocols were approved by the hospital’s ethics committee (approval numbers: 2018-92, 2025-080-02) and conformed to the principles set out in the World Medical Association Declaration of Helsinki and the Department of Health and Human Services Belmont Report.

### COPD-diabetic hyperglycemia comorbid mice model

Mice were purchased from GemPharmatech Co., Ltd (Nanjing, China). All animal experiments were performed under protocols approved by the Animal Care and Use Committee of The First Affiliated Hospital of Guangzhou Medical University (approval number: 20250029). Eight- to ten-week-old specific pathogen-free male C57BL/6 mice were used. All mice were housed on a regular 12 h light/dark cycle, room temperature: 20–25 °C, relative humidity: 50–70%.

We first established a mouse model of diabetic hyperglycemia by intraperitoneal injection of single doses of STZ (200 mg/kg of body weight) in 0.1 M citrate buffer (pH 4.5) and supplied with 10% sucrose water for 2 days. STZ is a small molecule that has been shown to selectively destroy pancreatic beta islet cells and induce diabetic hyperglycemia within mice (Suzuki et al, 2013; Lee et al, 2016; Deng et al, 2023). Animals with 6 h fasting blood glucose values higher than 15 mM were considered diabetic hyperglycemia. Three days later, we established a mouse model of COPD-like emphysema by intranasal instillation of 1.2 U porcine pancreatic elastase in 60 μl PBS once a week for four weeks (Liang et al, 2023; Xu et al, 2023; Hu et al, 2024). The diabetic state was maintained throughout the 4‑week COPD development period, as confirmed by weekly blood glucose monitoring. At the endpoint, successful comorbidity was validated by persistent hyperglycemia (≥15 mM), emphysematous changes (enlarged alveoli, increased mean linear intercept), and elevated AGEs levels in the airway.

Meanwhile, the mice were treated with 80 mg/kg body weight of aminoguanidine (AG) in drinking water daily (Gao et al, 2024), 3 mg/kg/d azeliragon (RAGE inhibitor) by oral gavage (Burstein et al, 2018; Zhou et al, 2025), or 10 mg/kg/d L6H21 (MD2 inhibitor) by oral gavage (Fang et al, 2015; Wang et al, 2020; Ge et al, 2024) until the study endpoint.

### Single-cell RNA sequencing (scRNA-seq)

Bronchial brushing samples were collected from three COPD patients without diabetes and two COPD patients with diabetes during bronchoscopy and immediately placed in ice-cold preservation buffer. Then, the samples were washed and filtered through a 70-μm cell strainer to remove mucus and obtain a single-cell suspension. Cell viability and concentration were assessed before proceeding. Using a platform such as 10x Genomics, single cells are encapsulated with barcoded beads in droplets for cell lysis and mRNA capture. Reverse transcription and cDNA amplification are performed to construct sequencing libraries, which are pooled and sequenced on an Illumina NovaSeq platform. Bioinformatics analysis included dimensionality reduction (PCA), clustering (UMAP), and cell type annotation were performed in Seurat (v4.3.0) using established marker genes: basal cells (*KRT5*, *KRT15*, *TP63*), secretory cells (*SCGB1A1*, *SCGB3A1*, *MUC5AC*, *MUC5B*), ciliated cells (*CIMIP1*, *PIFO*, *TPPP3*), macrophages (*CD68*, *MS4A7*), T cells (*CD2*, *CD3D*, *IL32*), NK cells (*NKG7*, *GNLY*), and ionocytes (*CFTR*, *FOXI1*, *ASCL3*). Differential gene expression and functional enrichment (GO and KEGG) were analyzed for each cell type. The clinical characteristics of the patients were provided in Table EV3.

### Air-liquid interface (ALI) culture system

ALI-cultured airway epithelial cells were derived from primary human bronchial epithelial progenitor cells isolated from fresh specimens of COPD patients. Briefly, human bronchial epithelial progenitor cells were expanded in PneumaCult™-Ex Plus Medium (Stemcell Technologies, Cat# 05040). At passage 2, 4 × 10⁴ cells/insert were seeded onto 0.4-μm-pore Transwell® Permeable Supports (Corning, Cat# 3470). Upon confluence (Day 0), apical medium was removed, and cultures were maintained in PneumaCult™-ALI Medium (Stemcell Technologies, Cat# 05001) for 28 days (medium changed every 2 days), as previously reported by our group (Peng et al, 2023; Qiu et al, 2024). At day 28 post-differentiation, ALI-cultured airway epithelium derived from the same donor in different Transwell inserts received bilateral application of treatments: high-glucose (osmotic control: equimolar mannitol), AGEs (control: equimolar BSA), and inhibitors (AG/RAGEi/MD2i with vehicle controls: PBS/DMSO). Prior to application, ALI-cultured airway epithelium underwent three 10-minute washes with pre-warmed PBS (37 °C) on the apical surface.

### IAV infection and detection

The IAV (A/Puerto Rico/8/1934 H1N1) was kindly provided by Professor Lei Wang, Guangzhou Medical University, Guangzhou, China. ALI-cultured airway epithelium on the apical chamber of the transwell inserts was infected with viral inoculum in PBS at an MOI of 0.5. The inoculated plates were incubated for 2 h at 37 °C with 5% CO_2_. At the end of the incubation, the inoculum was removed from the apical chamber. One day post-infection, the total RNA, including viral RNA, was extracted from the cell lysate and cell supernatant by using TRIzol reagent and the EZ-press Viral RNA Purification Kit (EZBioscience, Cat# EZB-VRN1), respectively. In murine experiments, each mouse received intranasal administration of 5 × 10^4^ PFU IAV in 60 μl PBS. Trachea and lung were harvested at 3 days post-infection, homogenized, and processed for RNA extraction using TRIzol reagent. To quantify IAV copy numbers, cDNA was subsequently generated using IAV-specific RT primer (uni-12: 5′-AGCAAAAGCAGG-3′) and monitored using a quantitative PCR assay for the IAV-*M1* gene. A plasmid containing the IAV-*M1* gene served as the quantification standard, enabling conversion of cycle threshold (CT) values to viral copy numbers via a standard curve generated from serial dilutions of the plasmid with known copy concentrations.

### siRNA transfection of airway epithelial cells in ALI

The cells were transfected according to the manufacturer’s protocol and a previous study (Yi et al, 2023) with minor modifications. Briefly, primary airway basal cells were transfected at ALI starting from day 5 post‑differentiation. siRNA was used at a final concentration of 160 nM in the basal chamber, containing 15 µL of siRNA‑mate plus (GenePharma, China, Cat# G04027). Transfections were repeated every three days for a total of 21 days. siRNAs targeting RAGE and MD2 were designed and constructed by GenePharma (Shanghai, China), and the siRNA‑targeted sequences are listed in the Reagents and Tools table.

### Cytospin preparation

Primary human airway epithelial cells were harvested from ALI culture via 0.25% trypsin-EDTA dissociation (37 °C, 10 min), and bronchoalveolar lavage fluid (BALF) samples were obtained from COPD patients. Cellular suspensions underwent centrifugation at 2000 rpm for 5 min and the pellets were fixed in 4% paraformaldehyde at room temperature for 10 min, followed by being washed 3 times with PBS for 5 min each time. Cytospin preparation (2 × 10^4^ cells/slide) was created by spinning for 5 min at 500 rpm on a Shandon Cytospin 4 Cytocentrifuge (Thermo Fisher Scientific). For cytomorphological analysis, standardized cytospin preparations were generated using 2 × 10⁴ cells per slide. Samples were loaded into Shandon Cytofunnels® and centrifuged at 500 rpm for 5 min using a Shandon Cytospin™ 4 Cytocentrifuge (Thermo Fisher Scientific) with a low-acceleration profile control to preserve cellular architecture for downstream staining protocols.

### Protein preparation and Western blot (WB) analysis

Total proteins were extracted by using lysis buffer supplemented with phosphatase and protease inhibitor cocktail. Protein extracts were subjected to SDS-PAGE and transferred to polyvinylidene difluoride (PVDF, Merck Millipore) membranes, which were then blocked with QuickBlock™ Blocking Buffer (Beyotime, Cat# P0252) for 15 min at room temperature and incubated with specific primary antibodies overnight at 4 °C, followed by being incubated with HRP-conjugated secondary antibodies for 1 h at room temperature. The band was visualized by chemiluminescence and quantified by ImageJ. WB results for proteins with similar molecular weights were obtained either by stripping and re‑probing the same membrane, or, when stripping was inefficient, by running the same samples on parallel gels.

### Co-Immunoprecipitation (Co-IP) and biotinylated protein pull-down assays

ALI-cultured airway epithelium treated with biotin label-AGEs was collected and lysed using Cell Lysis Buffer for Immunoprecipitation (Beyotime, Cat# P0013). For Co-IP, lysate was incubated with RAGE antibody or MD2 antibody with a volume ratio of 1:100 overnight at 4 °C, followed by the addition of 20 μL Protein A + G Magnetic Beads (Beyotime, Cat# P2108) with gentle agitation at 4 °C for 2 h. For biotinylated protein pull-down assays, lysate was incubated with Streptavidin Magnetic Beads (Beyotime, Cat# P2151) overnight at 4 °C. The beads were then washed with TBS three times and resuspended in 1×SDS sample buffer, followed by western blotting with a specific antibody against the AGEs, RAGE or MD2.

### Quantitative real-time PCR (qPCR)

Total RNA was extracted from ALI-cultured airway epithelium or bronchial tissue with TRIzol reagent (Invitrogen, Cat# 15596018) under the manufacturer’s instructions. Complementary DNA synthesis was performed using Hifair® Ⅲ 1st Strand cDNA Synthesis SuperMix (Yeasen, Cat# 11141ES60). Real-time PCR reactions were performed on a QuantStudio™ 7 Real-Time PCR System (Applied Biosystems) using Hieff UNICON^®^ qPCR SYBR Green Master Mix (Yeasen, #11199ES08). The primer sequences used for qPCR are listed in Table EV4. Data were normalized to the expression of reference gene (*ACTB*), and the 2^−ΔΔCt^ method was applied to quantify relative mRNA expression.

### Enzyme-linked immunosorbent assay (ELISA)

AGEs concentrations in bronchoalveolar lavage fluid (BALF) and ALI culture supernatant, as well as the levels of IL‑1β, TNF‑α, IL‑6, IFN‑β, CCL2, CXCL2, and CXCL3 in the supernatant of ALI culture, and the levels of IL‑1β, TNF‑α, CCL2, CXCL2, and CXCL3 in mouse BALF, were measured using commercial ELISA kits according to the manufacturer’s protocol. Absorbance was read at 450 nm on a Multiskan GO microplate reader (Thermo Fisher Scientific).

### Release of lactate dehydrogenase (LDH)

Airway epithelial injury in ALI culture was assessed using the LDH Cytotoxicity Detection Kit (Beyotime, Cat# C0017) to detect the release of LDH. Briefly, 120 µl of basolateral medium collected from ALI culture was mixed with 60 µl of reaction buffer. Samples were incubated for 30 min at room temperature (RT) in the dark and absorbance at 490 nm (reference wavelength: 600 nm) was measured using a Multiskan GO microplate reader (Thermo Fisher Scientific). A maximum LDH release control was included in each experiment, established by treating ALI-cultured airway epithelium with the provided lysis solution for 1 h at 37 °C prior to medium collection.

### Scanning electron microscopy (SEM)

Bronchial tissue specimens from COPD patients obtained via surgical resection were fixed in 2.5% glutaraldehyde, followed by post‑fixation in 1% osmium tetroxide. The samples were then dehydrated through a graded ethanol series, subjected to critical point drying or tert‑butanol freeze‑drying for thorough dehydration, and mounted onto aluminum stubs using double‑sided carbon tape. Finally, the specimens were sputter‑coated with a 5–10 nm gold or platinum layer and examined under an FEI Quanta 250 FEG scanning electron microscope.

### Immunofluorescent (IF) staining

Human Bronchial tissue sections, cytospin preparations from bronchoalveolar lavage fluid (BALF), and ALI-cultured cells were fixed with 4% formaldehyde. Subsequently, tissue samples were processed into paraffin-embedded blocks and sectioned, while BALF cells and ALI-cultured cells were deposited onto slides using Cytospin centrifugation to generate cell smears. Next, the slides underwent heat-induced antigen retrieval using citrate buffer (pH 9.0), were permeabilized with 0.5% Triton X-100, blocked with 10% goat serum and incubated with the specified primary antibodies overnight at 4 °C. Alexa Fluor^®^-conjugated secondary antibodies (Invitrogen; 1:500) were used and the nucleus was labeled with DAPI (1:1000). Fluorescence images were captured on a DM6 microscope (Leica), and mean fluorescence intensity (MFI) was quantified with ImageJ.

### Hematoxylin-Eosin staining

Bronchial tissues obtained via surgical resection from COPD patients (with/without diabetes) and PPE/STZ-modeled mice were fixed in 4% paraformaldehyde and then embedded in paraffin. Serial sections (4-μm thickness) were deparaffinized in xylene, rehydrated through a graded ethanol series, stained with Mayer’s hematoxylin for 8 min and eosin Y for 1 min, dehydrated in ascending ethanol concentrations, cleared in xylene, and mounted with synthetic resin.

### Statistical analysis

No specific inclusion/exclusion criteria were pre‑defined for statistical analysis, except for technical failures (e.g., failed assays and equipment malfunction). For animal experiments, mice were randomly assigned to treatment groups. Investigators were not blinded to group allocation during the experiments, but all quantitative analyses were performed using automated or objective quantitative methods to minimize subjective bias. Statistical analyses were conducted using GraphPad Prism 8.0 (GraphPad Software, Inc.). Data were tested for normal distribution with the Shapiro–Wilk test or the Kolmogorov–Smirnov test. For normally distributed data in ALI culture experiments, two-group comparisons employed paired *t*-test while multiple comparisons used RM one-way ANOVA with Dunnett’s or Fisher’s LSD post hoc test or RM two-way ANOVA with Bonferroni’s post hoc test; corresponding analyses for animal/human samples utilized unpaired *t*-test (two groups) and one-way ANOVA with Fisher’s LSD post hoc test (multiple groups). Non-normally distributed data of ALI culture experiments were analyzed with Wilcoxon signed-rank test (two groups) or Friedman with Dunn’s post hoc test (multiple groups), while animal/clinical data used the Mann–Whitney test (two groups) and Kruskal–Wallis with Dunn’s post hoc test (multiple groups). Categorical variables underwent Chi-square test and correlation was evaluated by Spearman’s test. Data were presented as mean ± SD and “*n*” denotes biological replicates unless otherwise noted. Sample sizes were determined based on the literature documentation of similar well-characterized experiments. *P* value less than 0.05 was considered statistically significant.

## Supplementary information

Appendix

Table EV1

Table EV2

Table EV3

Table EV4

Peer Review File

Source data Fig. 1

Source data Fig. 2

Source data Fig. 3

Source data Fig. 4

Source data Fig. 5

Expanded View Figures

## Data Availability

The single-cell sequencing data produced in this study have been deposited in the SRA database, under accession number PRJNA1490316 (https://www.ncbi.nlm.nih.gov/sra/PRJNA1490316). Informed consent was obtained for data deposition and sharing, and the data have been deposited in accordance with the relevant ethical approvals and applicable privacy requirements. The source data of this paper are collected in the following database record: biostudies:S-SCDT-10_1038-S44321-026-00502-9.
