# Supplementary material for: Blocking AGEs reduces airway epithelial viral susceptibility in diabetes-exacerbated COPD
Source: EMBO Mol Med. 2026 Aug 17;18(9):3745–66. doi: 10.1038/s44321-026-00502-9 (PMC13562547; doi:10.1038/s44321-026-00502-9)
Supplement: Supplementary file 1 [file 44321_2026_502_MOESM12_ESM.pdf]

Expanded View Figures

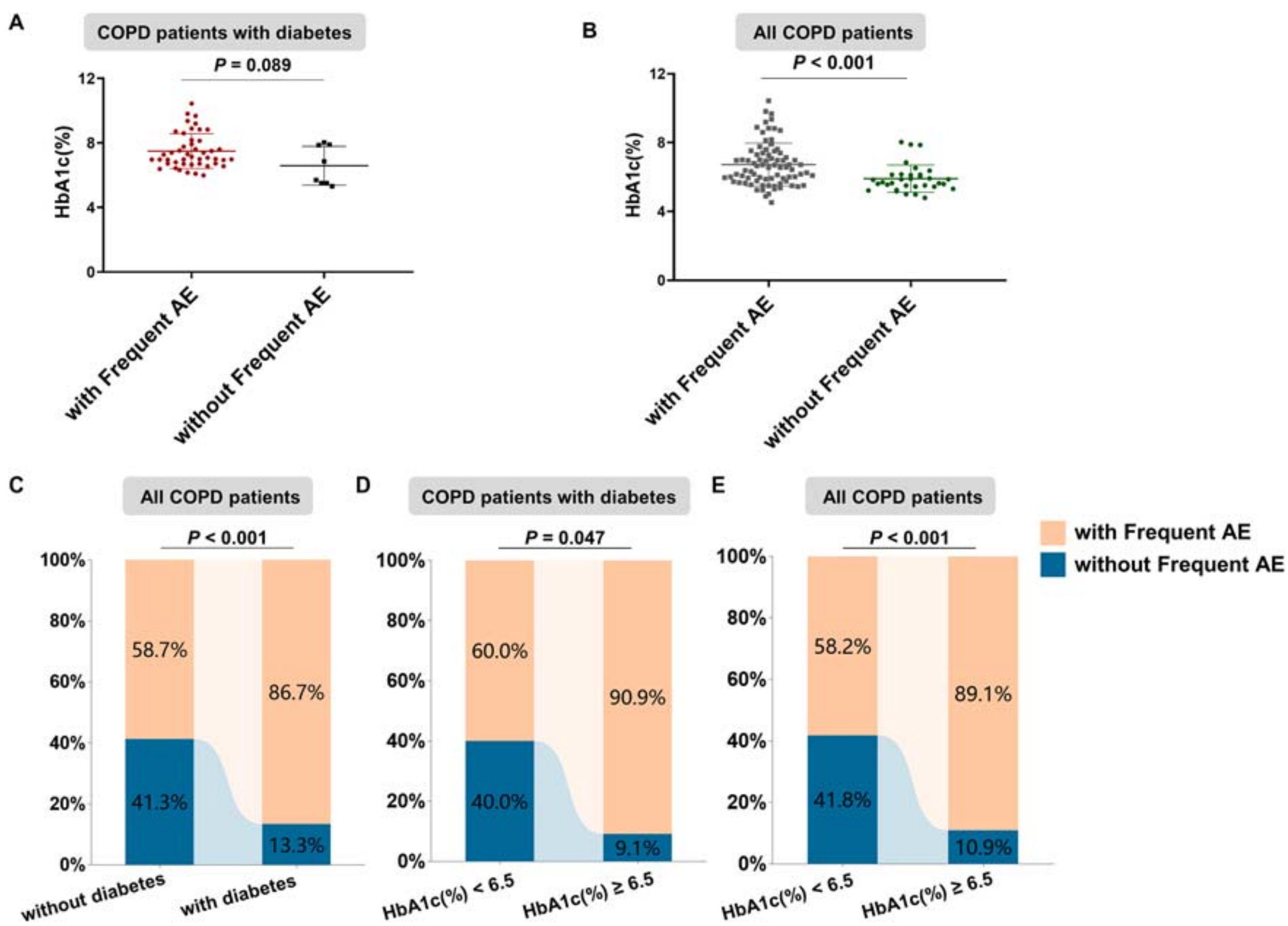

Figure EV1. HbA1c level correlates with frequent acute exacerbation (AE) in COPD Cohorts.

(**A**, **B**) HbA1c (%) level in the COPD patients with diabetes subgroup ($n = 60$) (**A**) and in the overall COPD cohort ($n = 123$, including non-diabetic subjects) (**B**), stratified by the presence or absence of frequent AE. (**C**) Proportion of patients with or without frequent AE in the overall COPD cohort stratified by those with or without diabetes. (**D**, **E**) Proportion of patients with or without frequent AE in the COPD patients with diabetes subgroup (**D**) and in the overall COPD cohort (**E**), stratified by HbA1c(%) < 6.5 vs. HbA1c(%) ≥ 6.5. Data are shown as Mean ± SD (**A**, B) or as percentage within each group (**C–E**). Statistical analyses were performed using the Mann–Whitney test (**A**, **B**), Chi-square with Yates' correction (**D**) or Chi-square (**C**, **E**). *n* indicates biological replicates.

 © The Author(s)

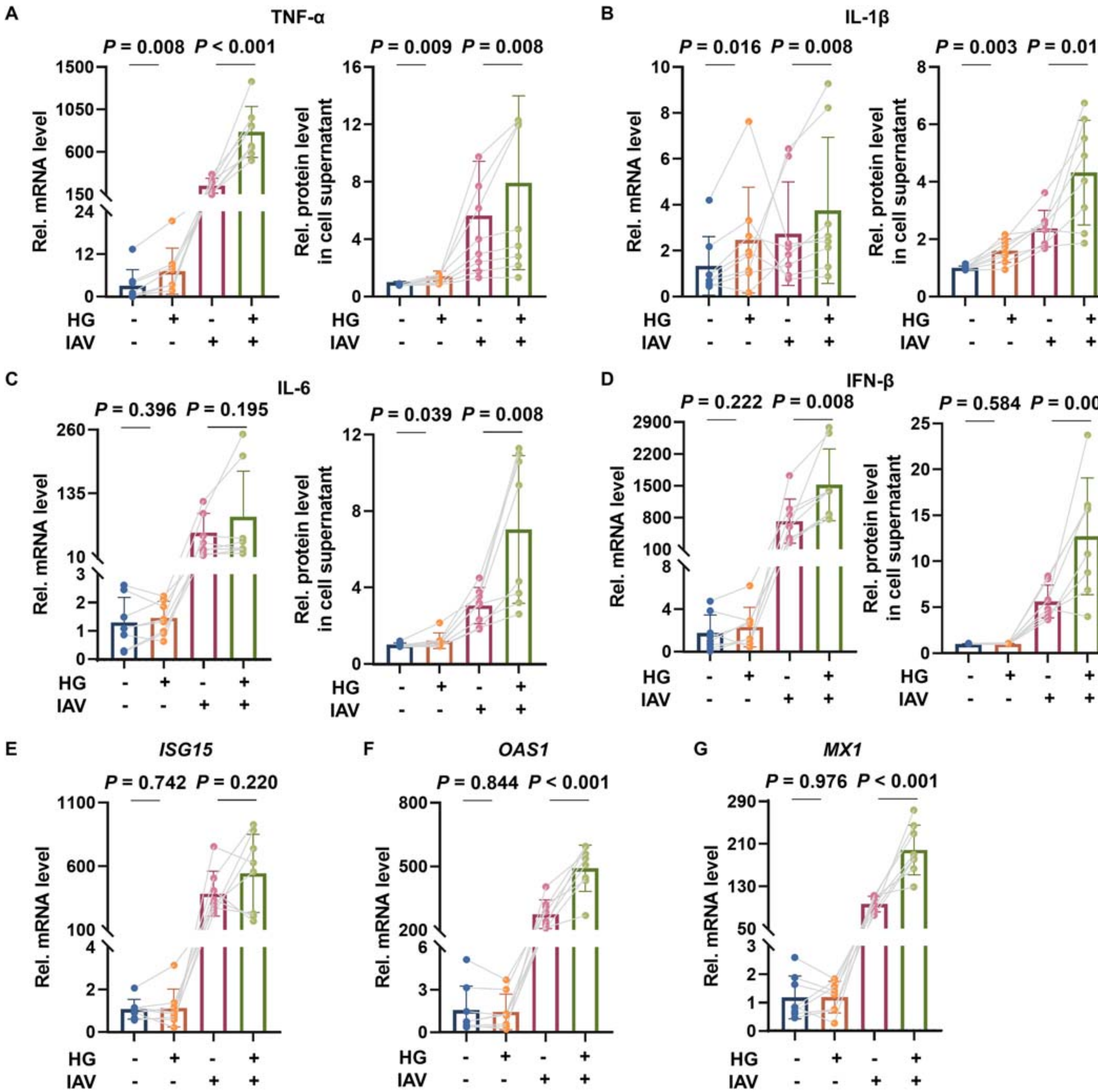

Figure EV2. High glucose (HG) potentiates IAV-mediated pro-inflammatory responses in airway epithelium derived from COPD.

qPCR (**A–D**, left panel; **E–G**) and ELISA (**A–D**, right panel) measurement of several cytokines and antiviral-related genes in ALI culture treated with or without 9 g/L HG for 3 days, followed by IAV infection (MOI = 0.5, 24 h) or mock infection. $n = 8$. Data were shown as Mean ± SD. The Wilcoxon signed-rank test was used for the following panels: **A** left panel (first two groups), A right panel (last two groups), **B** left panel, C left panel (last two groups), **C** right panel, **D** left panel (last two groups), **E** left panel (first two groups), and **F** left panel (first two groups). The paired t-test was used for all other comparisons. n indicates biological replicates. Related to Fig. 1.

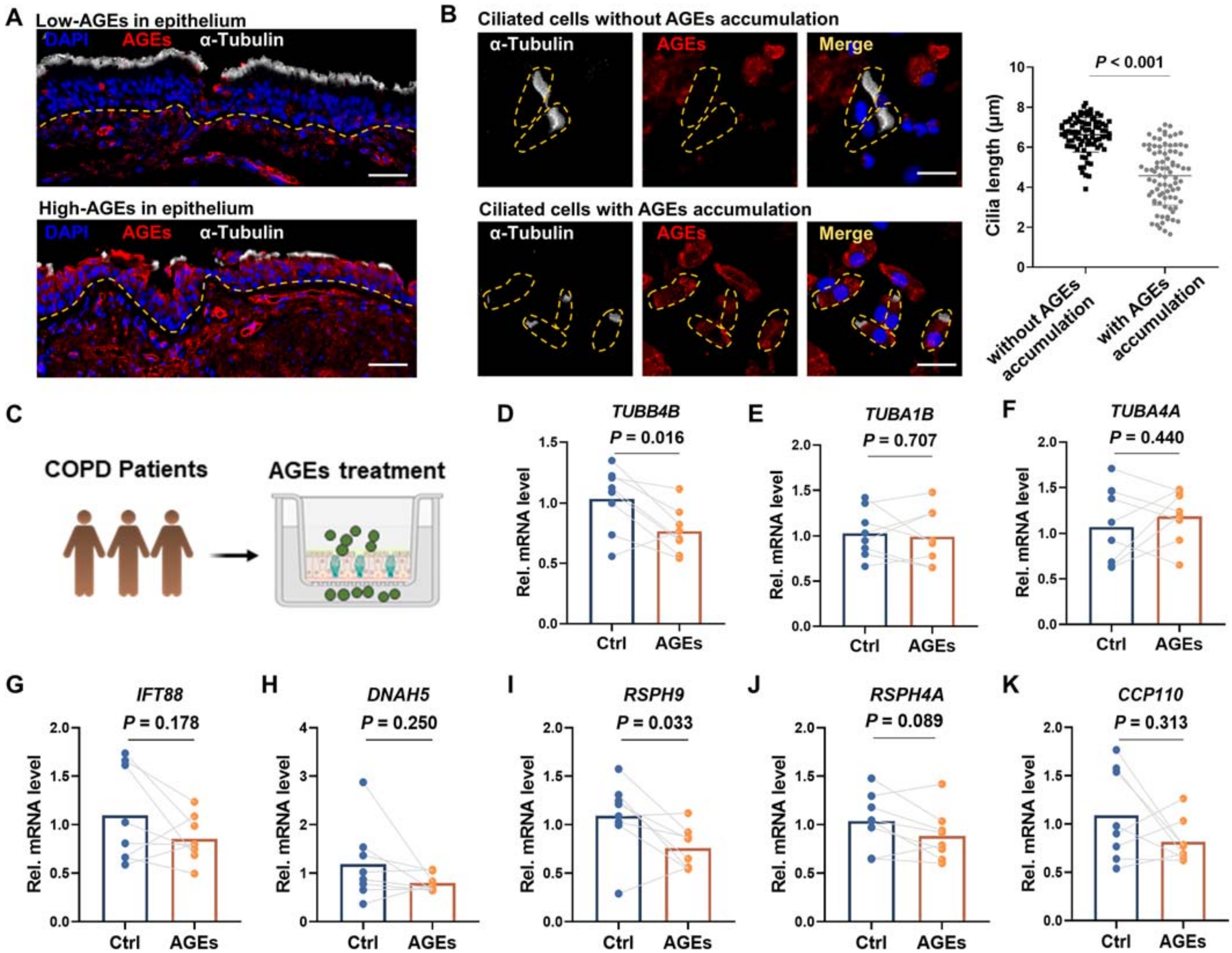

Figure EV3. AGEs exposure is associated with epithelial damage.

(**A**) Immunofluorescence staining image of airway cilia (α-Tubulin, white) in low-AGEs regions (upper panel, red) or high-AGEs areas (lower panel, red). Nuclei: DAPI (blue). The dashed line indicates the basal side of the airway mucosal epithelium. Representative images from three independent experiments are shown. Scale bar: 50 μm. (**B**) Representative immunofluorescence staining image of cilia (α-Tubulin, white) in BALF-cytospin cells without or with AGEs (red) accumulation. Yellow dashed borders outline individual ciliated epithelial cells. Nuclei: DAPI (blue). Scale bar: 10 μm. $n = 83$–84. (**C**) Schematic of exogenous AGEs exposure in ALI culture. (**D–K**) qPCR measurement of several cilia-related genes in ALI culture treated with or without 200 μg/mL AGEs for 1 day. $n = 8$. Data were shown as Mean ± SD and analyzed by unpaired *t*-test (**B**), paired *t*-test (**D–G**, **I**, **J**) or Wilcoxon signed-rank test (**H**, **K**). *n* indicates the number of cells (**B**) or biological replicates (**D–K**). Related to Fig. 2.

 © The Author(s)

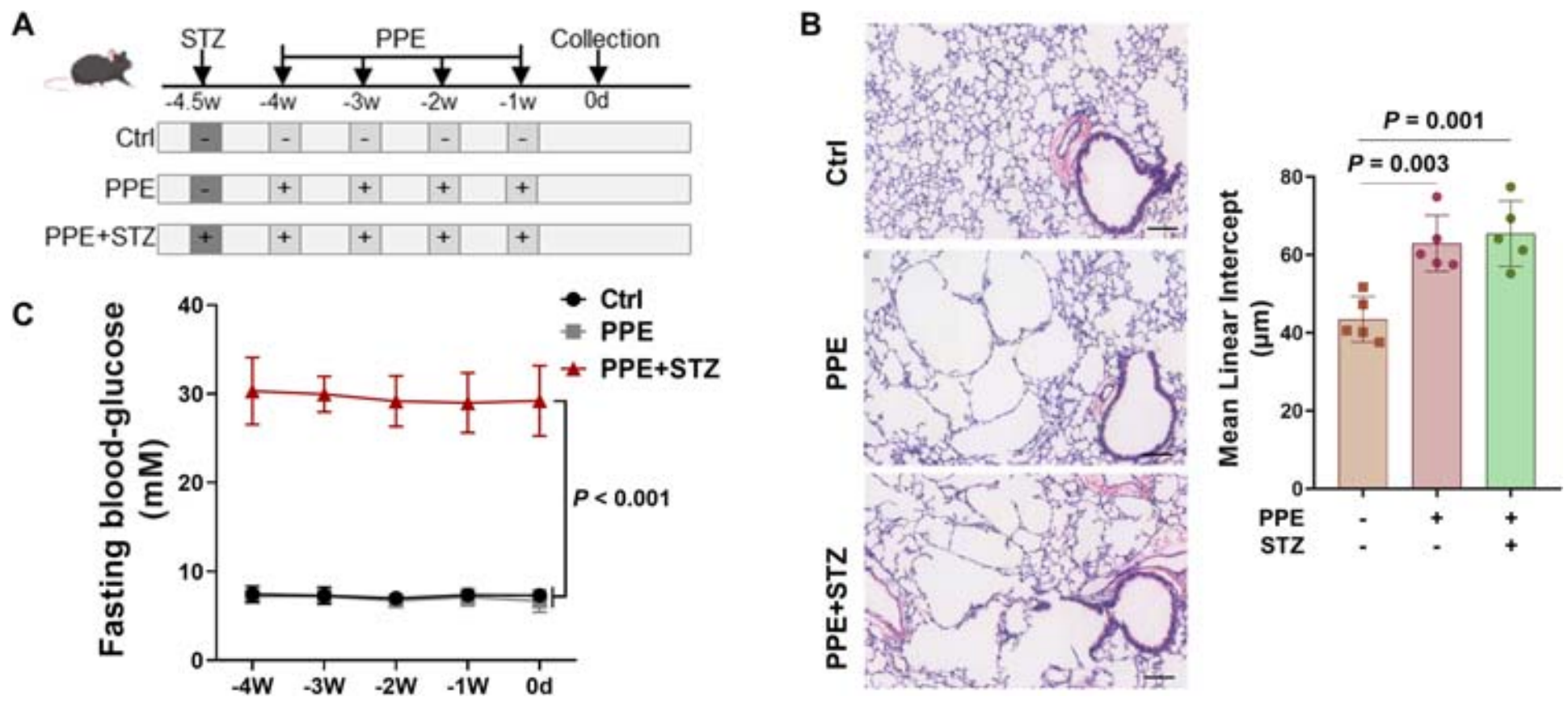

Figure EV4. Characterization of COPD-diabetes comorbid mouse model.

(**A**) Schematic showing C57BL/6 mice received PPE (porcine pancreatic elastase, intranasal, 1.2 U) + STZ (streptozotocin, intraperitoneal, 200 mg/kg) to establish a COPD-diabetes model. (**B**) Representative images of H&E-stained lung sections and the mean linear intercept (MLI) measurement. Scale bar: 100 μm. *n* = 5. (**C**) Fasting blood glucose levels of the COPD-diabetes mouse model. *n* = 5. Data were shown as Mean ± SD and analyzed by one-way ANOVA with Fisher's LSD post hoc test. *n* indicates biological replicates. Related to Fig. 3.

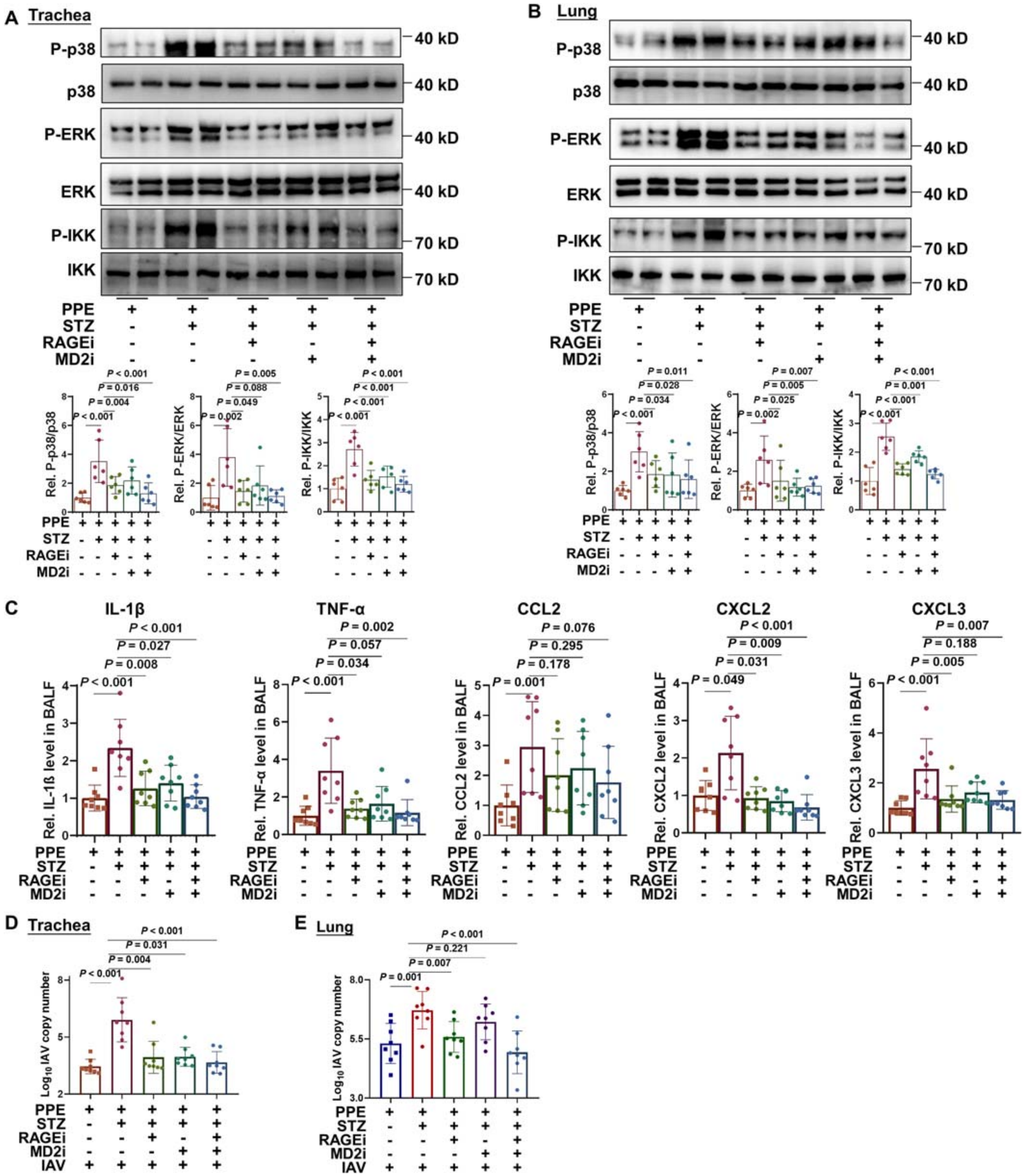

Figure EV5. RAGE and MD2 mediate diabetic hyperglycemia-induced pro-inflammatory signaling activation and viral susceptibility in COPD mice with diabetes.

(**A**, **B**) Western blot measurement of phospho-p38/ERK/IKK in trachea (**A**) and lung (**B**) of COPD-diabetes mice model treated with RAGEi or MD2i. $n = 6$. (**C**) ELISA measurement of several cytokines expression in BALF of COPD-diabetes mice model treated with RAGEi or MD2i. $n = 8$. (**D**, **E**) Quantification of IAV copy numbers in trachea (**D**) and lung (**E**) of COPD-diabetes mice model. $n = 8$. Data were shown as Mean ± SD and analyzed by one-way ANOVA with Fisher's LSD post hoc test (**A** left and right panel, **B**, **E**) or Kruskal–Wallis with Dunn's post hoc test (**A** middle panel, **C**, **D**). *n* indicates biological replicates. Related to Fig. 5.

 © The Author(s)
