# Supplementary material for: Blocking AGEs reduces airway epithelial viral susceptibility in diabetes-exacerbated COPD
Source: EMBO Mol Med. 2026 Aug 17;18(9):3745–66. doi: 10.1038/s44321-026-00502-9 (PMC13562547; doi:10.1038/s44321-026-00502-9)
Supplement: Supplementary file 2 [file 44321_2026_502_MOESM1_ESM.pdf]

Appendix for

Blocking AGEs Reduces Airway Epithelial Viral Susceptibility in Diabetes-Exacerbated COPD

Table of contents

Appendix Figure S1 ······························ 2

Appendix Figure S2 ······························ 3

Appendix Figure S3 ······························ 4

Appendix Figure S4 ······························ 5

Appendix Figure S5 ······························ 6

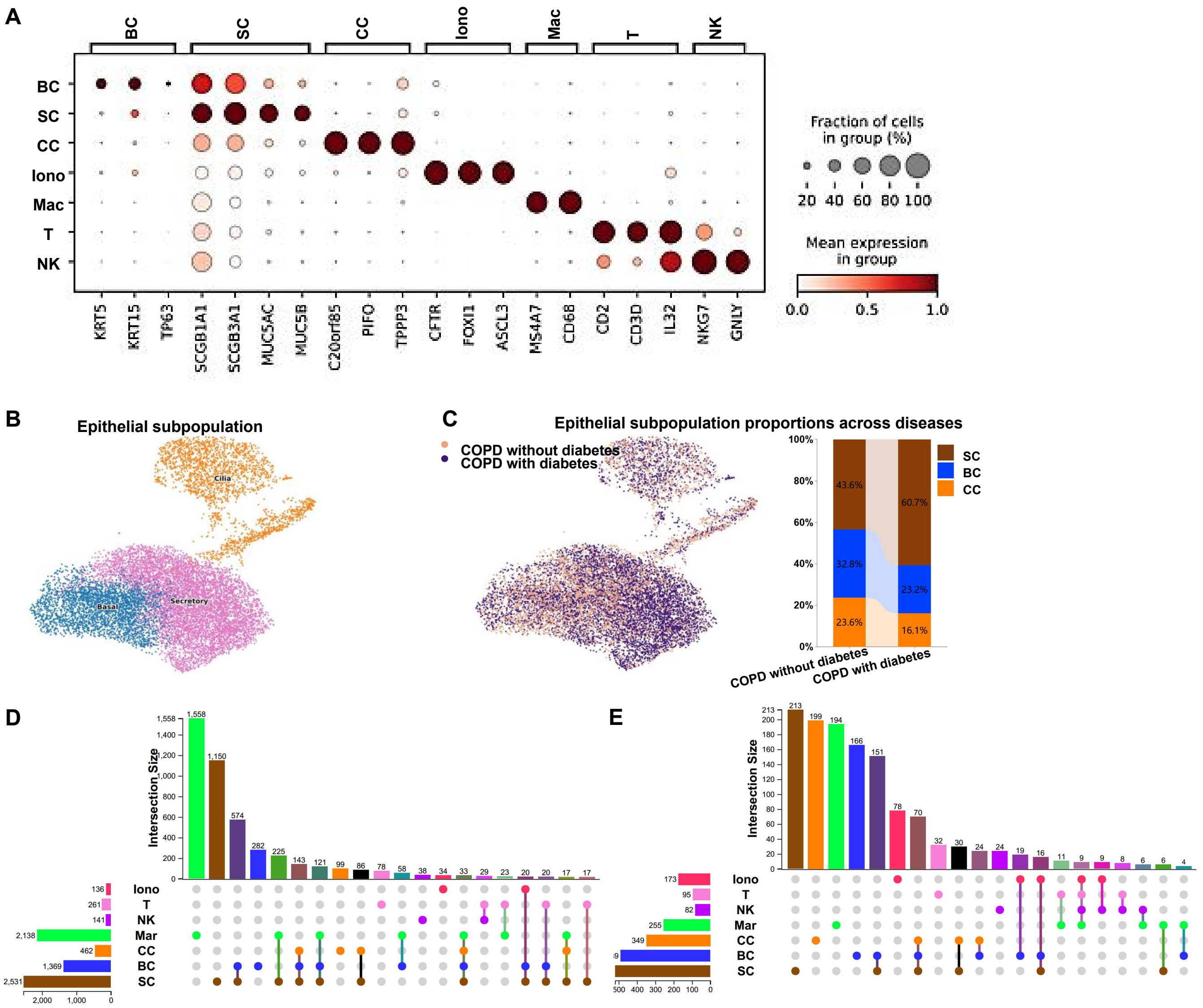

Appendix Figure S1. Single-cell transcriptomic characterization of airway epithelium in COPD with diabetes. (A) Dot plots of canonical markers defining cell clusters: BC, basal cells (*KRT5*, *KRT15*, *TP63*); SC, secretory cells (*SCGB1A1*, *SCGB3A1*, *MUC5AC*, *MUC5B*); CC, ciliated cells (*CIMIP1*, *PIFO*, *TPPP3*); Iono, ionocytes (*CFTR*, *FOXI1*, *ASCL3*); Mac, macrophages (*CD68*, *MS4A7*); T cells (*CD2*, *CD3D*, *IL32*); NK cells (*NKG7*, *GNLY*). **(B)** UMAP visualization of epithelial subsets re-clustered from Fig. 1B, including basal, secretory, ciliated cells. **(C)** Proportional quantification of epithelial subsets. **(D)** Upset plot illustrating the quantity of up-regulated differentially expressed genes (DEGs) and shared DEGs across cell types. **(E)** Upset plot showing the number of down-regulated DEGs and shared DEGs across cell types. SC, secretory cells; BC, basal cells; CC, ciliated cells; Mac, macrophages; NK, NK cells; T, T cells; Iono, ionocytes. **Related to Figure 1**.

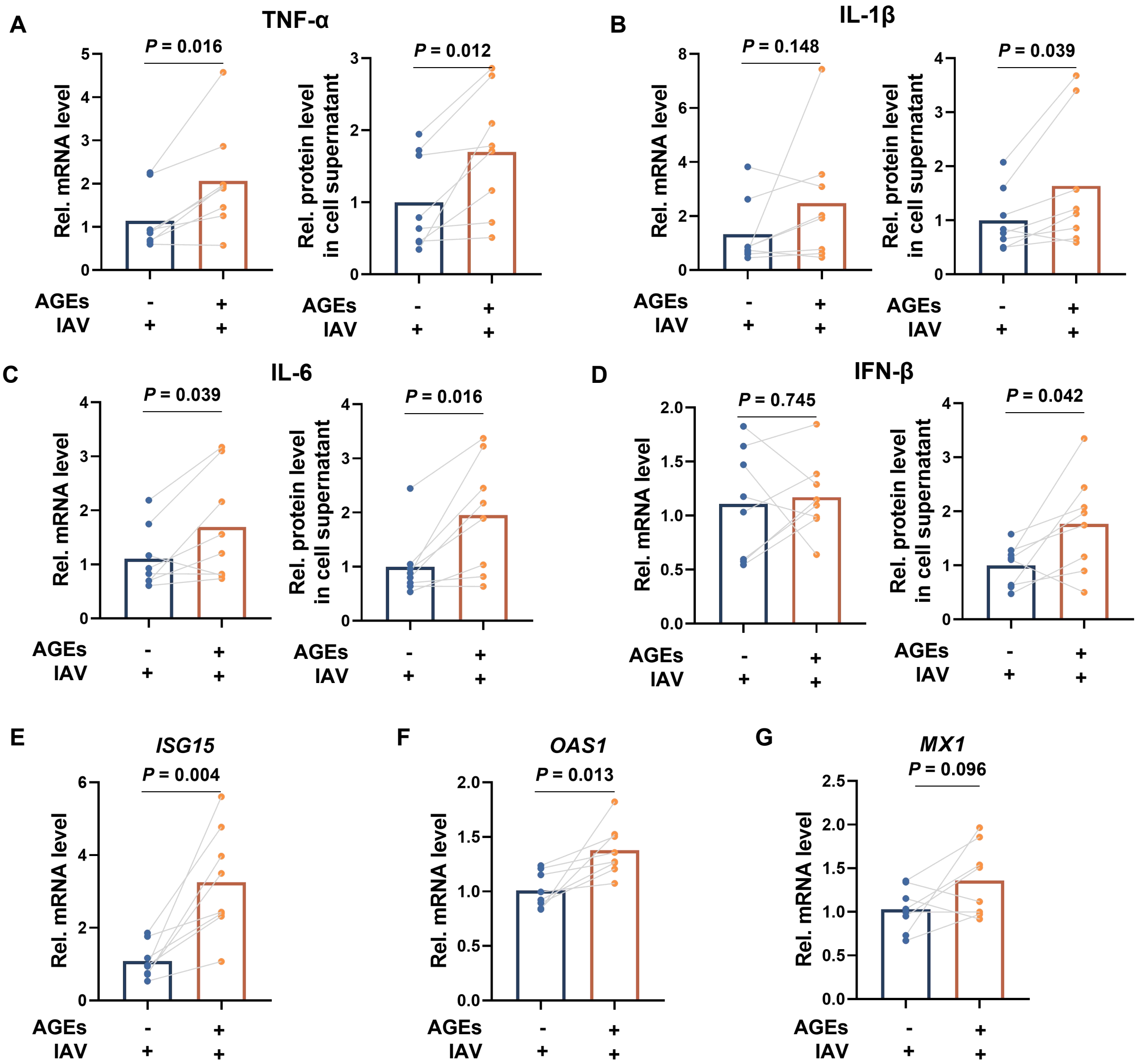

Appendix Figure S2. AGEs potentiate IAV-induced proinflammatory responses in airway epithelium derived from COPD. qPCR **(A-D, left panel, E-G)** and ELISA **(A-D, right panel)** measurement of several cytokines and antiviral-related genes in ALI culture treated with or without 200 µg/mL AGEs for 1 day followed by IAV infection (MOI = 0.5, 24 h). *n* = 8. Data are shown as *Mean* ± *SD and* analyzed by the Wilcoxon signed rank test (A left panel, B, C right panel) or paired t-test (A right panel, C left panel, D, E, F, G). *n* indicates biological replicates. **Related to Figure 2**.

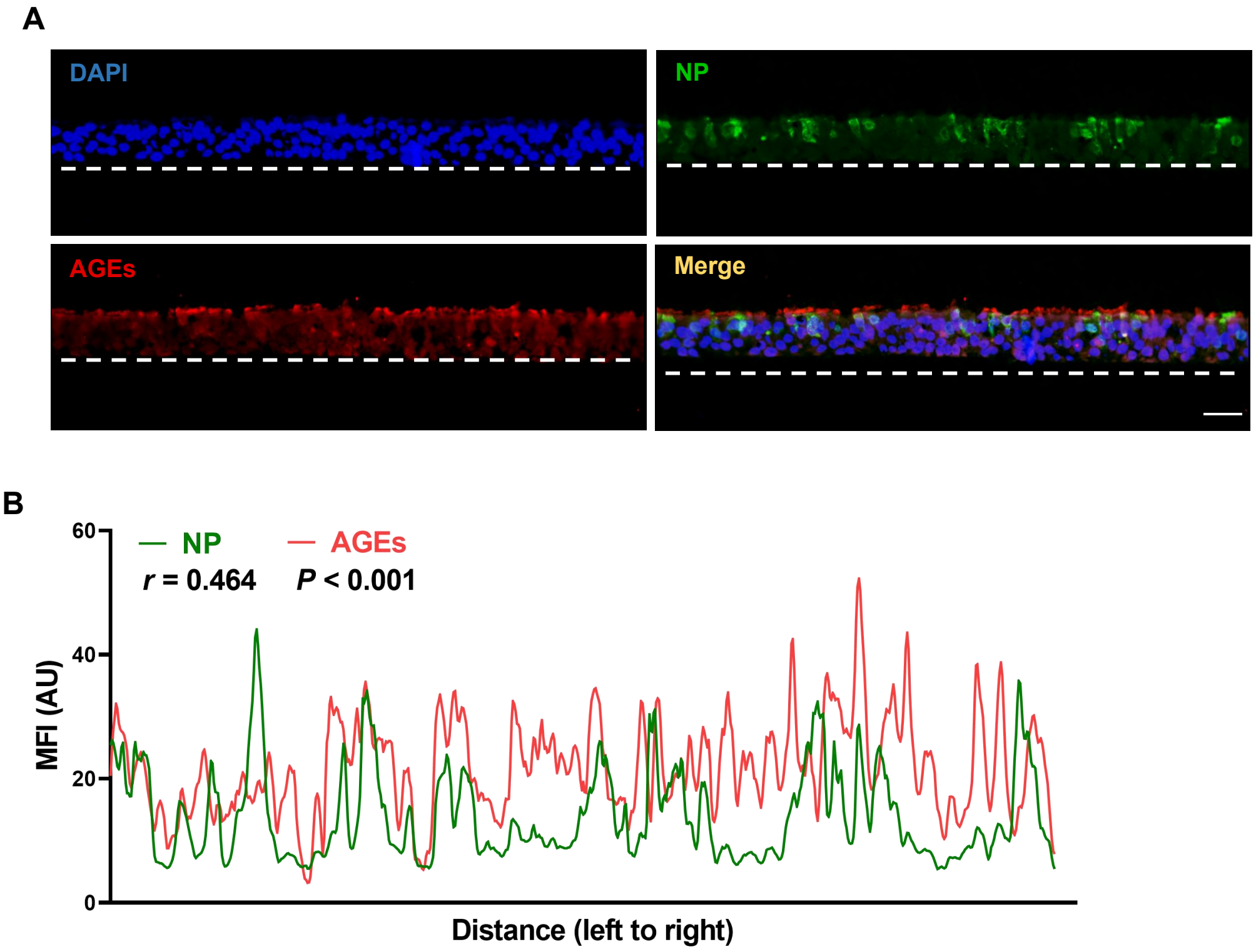

Appendix Figure S3. Spatial co-expression of AGEs and IAV in airway epithelium. (A) Representative immunofluorescence image of ALI culture (treated with 9 g/L HG for 3 days followed by infected IAV at MOI = 0.5 for 24 h) co-stained for AGEs (red) and IAV nucleoprotein (NP, green). Dashed line indicates the ALI basolateral side. Scale bar: 20 µm. **(B)** The line charts show the mean fluorescence intensity (MFI) of the distance in the images from left to right in arbitrary units (AU). Data are analyzed by Spearman's test. **Related to Figure 2**.

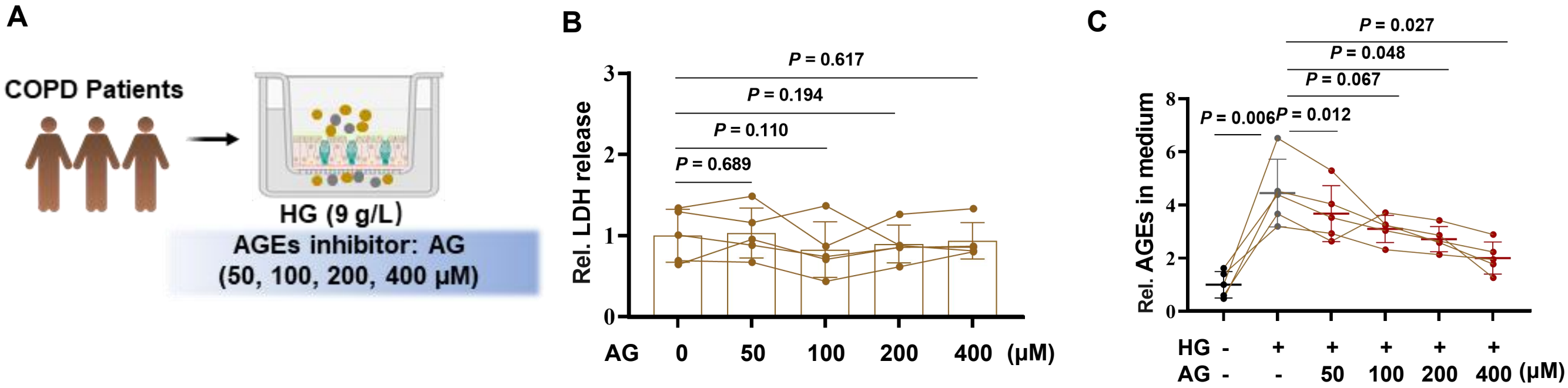

Appendix Figure S4. Biocompatibility and AGEs suppression efficacy of aminoguanidine (AG) in airway epithelium derived from COPD. (A) Schematic of AG treatment in ALI culture with AGEs exposure. **(B)** LDH release level in ALI culture exposed to AG across a concentration gradient (0-400 μM). *n* = 5. **(C)** ELISA measurement of AGEs in ALI supernatants after HG treatment accompanied different concentrations of AG (0-400 μM). *n* = 5. Data are shown as *Mean* ± *SD and* analyzed by Friedman with Dunn's *post hoc* test (B) or RM one-way ANOVA with Fisher's LSD *post hoc* test (C). *n* indicates biological replicates. **Related to Figure 3**.

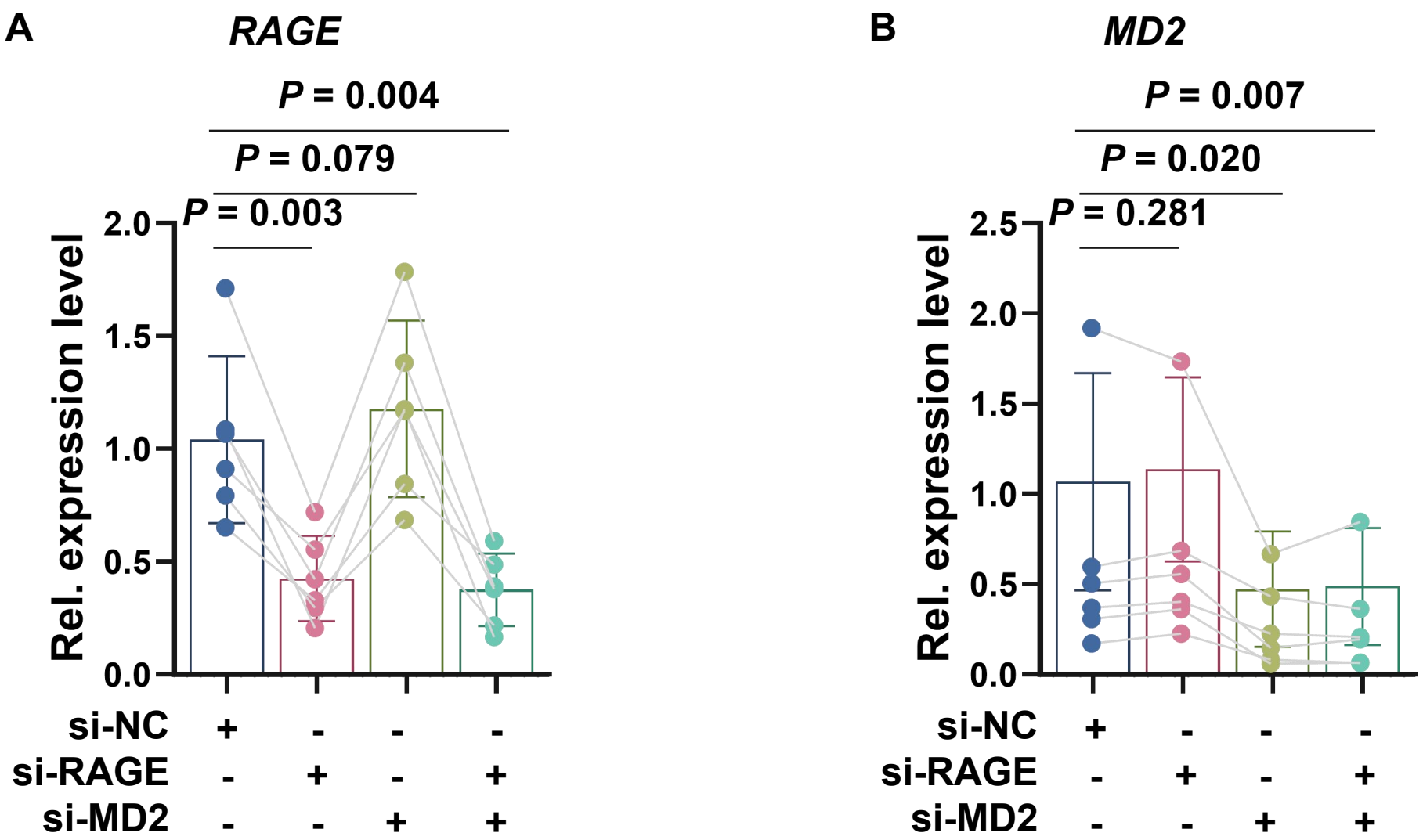

Appendix Figure S5. Validation of RAGE and MD2 siRNA knockdown efficiency in an ALI model of COPD with diabetes. qPCR measurement of RAGE **(A)** and MD2 **(B)** expression in ALI-cultured airway epithelium transfected with si-RAGE and/or si-MD2. n = 6. Data are shown as *Mean* ± *SD* and analyzed by RM one-way ANOVA with Fisher's LSD *post hoc* test. n indicates biological replicates. **Related to Figure 5**.
