# Supplementary material for: Blocking AGEs reduces airway epithelial viral susceptibility in diabetes-exacerbated COPD
Source: EMBO Mol Med. 2026 Aug 17;18(9):3745–66. doi: 10.1038/s44321-026-00502-9 (PMC13562547; doi:10.1038/s44321-026-00502-9)
Supplement: Supplementary file 3 [file 44321_2026_502_MOESM6_ESM.pdf]

EMBO Molecular Medicine

Blocking AGEs Reduces Airway Epithelial Viral Susceptibility in Diabetes-Exacerbated COPD

Zhenfeng Chen, Yueying Qin, Bingqi Lin, Linle Jiang, Zhencheng Feng, Shiying Chen, Zuodong Ye, Min Zhou, Jia Li, De Yun Wang, Nanshan Zhong, Panpan Hou, Ziqing Zhou, Lei Wang, and Yang Peng

Corresponding authors: Yang Peng (2023991035@gzhmu.edu.cn) , Lei Wang (leiwang2021@gzhmu.edu.cn), Ziqing Zhou (zzq@gird.cn), Panpan Hou (2024991003@gzhmu.edu.cn)

Review Timeline:

Submission Date:	3rd Dec 25
Editorial Decision:	15th Jan 26
Revision Received:	14th May 26
Editorial Decision:	17th Jun 26
Revision Received:	26th Jun 26
Accepted:	7th Jul 26

Editor: Lise Roth

Transaction Report:

(Note: With the exception of the correction of typographical or spelling errors that could be a source of ambiguity, letters and reports are not edited. Depending on transfer agreements, referee reports obtained elsewhere may or may not be included in this compilation. Referee reports are anonymous unless the Referee chooses to sign their reports.)

15th Jan 2026

Dear Dr. Peng,

Thank you for submitting your manuscript to EMBO Molecular Medicine, and please accept my apologies for the delay in getting back to you as I was waiting for the report from referee #2 before making a decision. However, referee #2 has not yet gotten back to us, and as both referees #1 and #3 make similar recommendations, we would like to make a decision now to avoid further delay in the process. If referee #2 provides a report, we will send it to you on the understanding that we will not ask you to carry out any extensive experiments beyond those requested in the attached reports from referees #1 and #3. As you will see below, the referees recognize the interest of the study and generally support the publication of your work, provided appropriate revisions.

Addressing the reviewers' concerns in full will be necessary for further considering the manuscript in our journal, and acceptance of the manuscript will entail a second round of review. EMBO Molecular Medicine encourages a single round of revision only and therefore, acceptance or rejection of the manuscript will depend on the completeness of your responses included in the next, final version of the manuscript. For this reason, and to save you frustration at the end, I would strongly discourage you from returning an incomplete revision.

If you would like to discuss further the points raised by the referees, I am available to do so via email or video. Let me know if you are interested in this option.

We are expecting your revised manuscript within three to four months, if you anticipate any delay, please contact us.

When preparing your revised manuscript, please refer to our guidelines: https://link.springer.com/journal/44321/submission-guidelines#cms-Revised-submissions. We perform an initial quality control of all revised manuscripts before re-review; failure to include requested items will delay the evaluation of your revision.

We require:

1) A .docx formatted version of the manuscript text (including legends for main figures, EV figures and tables). Please make sure that the changes are highlighted to be clearly visible.

2) Individual production quality figure files as .eps, .tif, .jpg (one file per figure). For guidance, download the 'Figure Guide PDF': https://media.springernature.com/original/springer-cms/rest/v1/content/27825798/data/v1.

3) A .docx formatted letter INCLUDING the reviewers' reports and your detailed point-by-point responses to their comments. As part of the EMBO Press transparent editorial process, the point-by-point response is part of the Review Process File (RPF), which will be published alongside your paper.

4) A complete author checklist, which you can download from our author guidelines. Please insert information in the checklist that is also reflected in the manuscript. The completed author checklist will also be part of the RPF.

5) Please note that all corresponding authors are required to supply an ORCID ID for their name upon submission of a revised manuscript.

6) It is mandatory to include a 'Data Availability' section after the Materials and Methods. Before submitting your revision, primary datasets produced in this study need to be deposited in an appropriate public database, and the accession numbers and database listed under 'Data Availability'. Please remember to provide a reviewer password if the datasets are not yet public.

In case you have no data that requires deposition in a public database, please state so in this section. Note that the Data Availability Section is restricted to new primary data that are part of this study.

7) For data quantification: please specify the name of the statistical test used to generate error bars and P values, the number (n) of independent experiments (specify technical or biological replicates) underlying each data point and the test used to calculate p-values in each figure legend. The figure legends should contain a basic description of n, P and the test applied. Graphs must include a description of the bars and the error bars (s.d., s.e.m.).

8) At EMBO Press we ask authors to provide source data for the main manuscript figures. You will receive a separate email with instructions for providing source data with your revised manuscript, including how to upload and organize the files.

9) Our journal encourages inclusion of *data citations in the reference list* to directly cite datasets that were re-used and obtained from public databases. Data citations in the article text are distinct from normal bibliographical citations and should

directly link to the database records from which the data can be accessed. In the main text, data citations are formatted as follows: "Data ref: Smith et al, 2001" or "Data ref: NCBI Sequence Read Archive PRJNA342805, 2017". In the Reference list, data citations must be labeled with "[DATASET]". A data reference must provide the database name, accession number/identifiers and a resolvable link to the landing page from which the data can be accessed at the end of the reference.

10) We replaced Supplementary Information with Expanded View (EV) Figures and Tables that are collapsible/expandable online. A maximum of 5 EV Figures can be typeset. EV Figures should be cited as 'Figure EV1, Figure EV2" etc... in the text and their respective legends should be included in the main text after the legends of regular figures.

- For the figures that you do NOT wish to display as Expanded View figures, they should be bundled together with their legends in a single PDF file called *Appendix*, which should start with a short Table of Content. Appendix figures should be referred to in the main text as: "Appendix Figure S1, Appendix Figure S2" etc.

- Additional Tables/Datasets should be labeled and referred to as Table EV1, Dataset EV1, etc. Legends have to be provided in a separate tab in case of .xls files. Alternatively, the legend can be supplied as a separate text file (README) and zipped together with the Table/Dataset file.

11) The paper explained: EMBO Molecular Medicine articles are accompanied by a summary of the articles to emphasize the major findings in the paper and their medical implications for the non-specialist reader. Please provide a draft summary of your article highlighting
- the medical issue you are addressing,
- the results obtained and
- their clinical impact.

This may be edited to ensure that readers understand the significance and context of the research. Please refer to any of our published articles for an example.

12) Author contributions: You will be asked to provide CRediT (Contributor Role Taxonomy) terms in the submission system. These replace a narrative author contribution section in the manuscript.

13) A Conflict of Interest statement should be provided in the main text.

14) Every published paper includes a 'Synopsis' to further enhance discoverability. Synopses are displayed on the journal webpage and are freely accessible to all readers. They include a short stand first (maximum of 300 characters, including space) as well as 2-5 one-sentences bullet points that summarizes the paper. Please write the bullet points to summarize the key NEW findings. They should be designed to be complementary to the abstract - i.e. not repeat the same text. We encourage inclusion of key acronyms and quantitative information (maximum of 30 words / bullet point). Please use the passive voice. Please attach these in a separate file or send them by email, we will incorporate them accordingly.

Please also suggest a striking image or visual abstract to illustrate your article as a PNG file 550 px wide x 300-600 px high.

15) Include a Reagents and Tools Table as part of the Methods section, which can be downloaded from our author guidelines.

16) As part of the EMBO Publications transparent editorial process initiative (see our Editorial at http://embomolmed.embopress.org/content/2/9/329), EMBO Molecular Medicine will publish online a Review Process File (RPF) to accompany accepted manuscripts.
In the event of acceptance, this file will be published in conjunction with your paper and will include the anonymous referee reports, your point-by-point response and all pertinent correspondence relating to the manuscript. Let us know whether you agree with the publication of the RPF and as here, if you want to remove or not any figures from it prior to publication.
Please note that the Authors checklist will be published at the end of the RPF.

EMBO Molecular Medicine has a "scooping protection" policy, whereby similar findings that are published by others during review or revision are not a criterion for rejection. Should you decide to submit a revised version, I do ask that you get in touch after three months if you have not completed it, to update us on the status.

I look forward to receiving your revised manuscript.

Yours sincerely,

Lise Roth

Lise Roth, PhD

Senior Editor
EMBO Molecular Medicine

***** Reviewer's comments *****

Referee #1 (Comments on Novelty/Model System for Author):

The sample collection from patients with "DM" is unclear, "DM" is a historical abbreviation and patient samples should be T1D/T2D or hyperglycemia

Referee #1 (Remarks for Author):

This paper shows that diabetic hyperglycemia drives airway epithelium cell injury and viral susceptibility in COPD. Authors have proven their hypothesis with multiple cellular models, patient samples and a mouse model of combined diabetes and COPD. Experiments are well performed. I only have some questions about the biological term DB and the sample collection from patients.

1) "COPD patients with DM exhibit increased prevalence of frequent acute exacerbation."
It should always be phrased to what is compared to (i.e. without DM)
"DM" should not be used- it is a historical abbreviation, please either use T1D/T2D or "diabetes"?

2) The DM group is not clear to me
"The diagnosis of COPD and DM was made based on the Global Initiative for Chronic Obstructive Lung Disease (GOLD) guidelines and American Diabetes Association (ADA)"
This is relevant, but it must be explained which measures were used to define the diseases, especially for "DM", the ADA has different measures for T1D & T2D
It looks like this correlation is dependent on hyperglycemia, i.e. HbA1c as there is a correlation between AE & HbA1C
The authors should make a threshold of elevated HbA1C defined as >6.5 and <6.5 to see whether the correlation is dependent on diabetes diagnosis or hyperglycemia, i.e. uncontrolled diabetes. Authors should also include separate correlations for T1D and T2D.
That means, the categories are very confusing. Either you do groups of hyperglycemia, i.e. HbA1c>6
or "diabetes": T1D & T2D

3) The phrase "diabetic COPD patients" should not be used, rather patient with T1D/ patient with T2D

4) Authors "demonstrated significant enrichment of multiple viral infection pathways, suggesting altered antiviral defense capacity in COPD patients with DM compared with those without DM".
The question is whether this increased antiviral defense observed in "DM" is really the reason for severe COPD or whether patients with T1D and T2D anyway have a basally elevated antiviral defense gene expression, also in lung epithelial, and this is independent of COPD; this needs to be checked by including control patients with "DM" without COPD.

5) Figure S3, for the cytokine PCR, please add all controls from Fig.1I (HG without virus) to see whether HG alone increases inflammation markers

6) From Fig.1J one cannot see increased viral replication in glucose-exposed epithelium

7) The blots should also show samples from "DM". What was the "DM" sample? High HbA1c?

8) Blot in Fig.5B, please remove the dotted lines, as there is no lane/blot separation.

Referee #3 (Comments on Novelty/Model System for Author):

The combination of human patient data, in vitro air-liquid interface cultures, and mouse is adequate for the studies. I have suggested additional experiments to be performed with the mouse model.

Referee #3 (Remarks for Author):

Review of the EMMM-2025-23076 by Chen et al.,

Targeting Diabetes-Exacerbated COPD Progression: Blocking AGEs to Rescue Viral Susceptibility in the Airway Epithelium

Individuals with diabetes have accelerated COPD progression, but the underlying mechanisms are poorly understood. In this study, Chen et al use scRNA-seq of airway epithelium to identify transcriptional alterations in diabetic versus non-diabetic COPD patients, focusing on host-defense pathways to viral infection, a key cause of COPD exacerbations. They found that hyperglycemia enhanced influenza virus susceptibility in both COPD-derived epithelial cells and diabetic COPD mice, suggesting a compromised airway defense. Furthermore, hyperglycemia promotes advanced glycation end product (AGEs) accumulation in the airway epithelium that correlates with infection severity. AGEs activate p38/ERK/IKK proinflammatory signaling via RAGE and MD2 receptors, leading to epithelial injury and heightened viral susceptibility. Inhibition of AGEs formation with aminoguanidine significantly reduced viral load. The authors concluded that the hyperglycemia-AGEs-RAGE/MD2 axis increases viral susceptibility in diabetic COPD patients. Overall, this is an extensive characterization of the contribution of AGEs to the pathogenesis of diabetic COPD. The manuscript is well written. In general, various conclusions are supported by extensive amounts of convincing experimental data. One problem with this study is that among the diabetics, the authors did not distinguish between type 1 versus type 2 diabetes, which should be clearly indicated in patient profiles, and may help to stratify type 1 vs. type 2 associated COPD phenotypes with regard to respiratory AGEs. Also, the deleterious effects of AGEs are well known and have been widely studied. The relative importance of RAGE vs. MD2 induced by diabetic COPD AGEs needs to be established. Whether or not AGEs in diabetic COPD-derived AGEs activate the non-canonical pathway (e.g., TGFβ2, ERK, AKT, and p38 MAPK) signaling is not addressed at all. A few additional minor suggestions are provided to further improve the manuscript.

Major comments:
1. Introduction, Lines 63-66. It will be useful to briefly describe the structural and functional defects of airway epithelial cells in COPD patients that may increase susceptibility to microbial infections, including the compromised barrier function.
2. Material and Methods, Lines 123-130. The description provided on the diabetic COPD mouse model is not clear. Please clearly indicate that diabetic mice were treated with porcine pancreatic elastase. Since elastase treatment requires 4 weeks, how was the diabetic state maintained in these mice?
3. Results, triplicate of histopathology, immunofluorescence images of ALI culture labeling, and western blots, as well as their quantification (e.g., with ImageJ, etc), need to be provided for all figures.
4. Results, Table S1. A major problem with this study is that among the diabetics, the authors did not distinguish between type 1 versus type 2 diabetes, which should be clearly indicated in patient profiles in Table S1, and may help to stratify type 1 vs. type 2 associated COPD phenotypes with regard to respiratory AGEs.
5. Fig. 1G-1J. 28 days are inadequate to allow full differentiation of ALI culture, where ~42 days are typically needed.
6. Figs. S3, S4, S5, 4G, and 5C. ELISA assays should be performed to support the qPCR-based expression of cytokines and anti-viral genes.
7. Fig. 5. The studies with RAGE and MD2 inhibitors and their impact on the expression of AGEs, anti-viral genes and cytokines, and viral loads need to be authenticated in the diabetic COPD mice.
8. In addition to inhibitors, the importance of RAGE and MD2 induced by diabetic COPD AGEs needs to be confirmed with siRNA studies.
9. The relative importance of RAGE vs. MD2 induced by diabetic COPD AGEs needs to be established.
10. How AGEs in diabetic COPD-derived AGEs in activating the non-canonical pathway (e.g., TGFβ2, ERK, AKT, and p38 MAPK) signaling is not addressed at all.

Minor comments
1. Introduction. Lines 85-86. ALI culture should be classified as in vitro, not ex vivo, which is typically reserved for organ cultures such as precision-cut lung slices.
2. Materials and Methods. Lines 109-115. The two sentences here sound redundant and need to be combined and rephrased.
3. Results, Lines 178-179. Please rephrase. The epithelial barrier is one of the primary airway defenses, but there are many other important ones, such as alveolar macrophages, surfactant lipid and proteins, antimicrobial peptides, etc.
4. Fig. S8, please define PPE and STZ in the legend.
5. Lines 303-304. Please provide references for AGEs interaction with RAGE and MD2.

Referee #1 (Comments on Novelty/Model System for Author):

The sample collection from patients with "DM" is unclear, "DM" is a historical abbreviation and patient samples should be T1D/T2D or hyperglycemia

Response: We apologize for the lack of clarity and thank you for the suggestion. "DM" in our original manuscript referred to "diabetes" and we have used the general term "diabetes" to replace "DM" in the revised manuscript. We collected samples from patients with "diabetes" according to the diagnosis criteria based on American Diabetes Association (ADA), i.e., meeting any one of the following four diagnostic thresholds: A1C ≥6.5%, fasting plasma glucose ≥126 mg/dL, 2-hour plasma glucose during OGTT ≥200 mg/dL, or random plasma glucose ≥200 mg/dL accompanied by classic hyperglycemic symptoms. These diagnostic criteria are identical for all types of diabetes, including type 1 (T1D), type 2 (T2D), gestational diabetes, and other specific types (PMID: 30559228; PMID: 41358893). Therefore, we did not strictly distinguish between T1D and T2D in our patient cohort in our original manuscript.

We have also re-examined the clinical information of the enrolled patients and found that all diabetic cases had type 2 diabetes (T2D); no type 1 diabetes cases were present in the cohort. Accordingly, we have also clarified that all diabetic patients in our cohort had type 2 diabetes (T2D) in "Clinical data collection" and "Ethics for human sample collection" subsections under the Methods section. We also acknowledge that the absence of type 1 diabetes (T1D) in our patient cohort is a limitation of this study. To address this, we have added a statement in the Discussion section (Page 26, Lines 629-635) clarifying the limitation with the description "all diabetic patients in our cohort had T2D, with no T1D cases identified; therefore, we did not stratify our analysis by diabetes type. Nevertheless, although the etiologies of T1D and T2D differ, both conditions converge on chronic hyperglycemia, which is the key driver of diabetic complications. Moreover, AGEs, the central molecule of this study and a key downstream product of hyperglycemia, are elevated in both types of diabetes, suggesting that similar findings may occur in both T1D and T2D populations."

We thank you again for pointing this out and hope these clarifications address the concern.

Referee #1 (Remarks for Author):

This paper shows that diabetic hyperglycemia drives airway epithelium cell injury and viral susceptibility in COPD. Authors have proven their hypothesis with multiple cellular models, patient samples and a mouse model of combined diabetes and COPD. Experiments are well performed. I only have some questions about the biological term DB and the sample collection from patients.

1) "COPD patients with DM exhibit increased prevalence of frequent acute exacerbation."
It should always be phrased to what is compared to (i.e. without DM). "DM" should not be used- it is a historical abbreviation, please either use T1D/T2D or "diabetes"?

Response: As mention above, we have replaced "DM" with "diabetes" throughout the manuscript. Thank you for pointing it out.

2) The DM group is not clear to me.
"The diagnosis of COPD and DM was made based on the Global Initiative for Chronic Obstructive Lung Disease (GOLD) guidelines and American Diabetes Association (ADA)".

This is relevant, but it must be explained which measures were used to define the diseases, especially for "DM", the ADA has different measures for T1D & T2D.

It looks like this correlation is dependent on hyperglycemia, i.e. HbA1c as there is a correlation between AE & HbA1C. The authors should make a threshold of elevated HbA1C defined as >6.5 and <6.5 to see whether the correlation is dependent on diabetes diagnosis or hyperglycemia, i.e. uncontrolled diabetes. Authors should also include separate correlations for T1D and T2D.

That means, the categories are very confusing. Either you do groups of hyperglycemia, i.e. HbA1c>6 or "diabetes": T1D & T2D.

Response: We thank you for this careful critique and apologize for the confusion caused by our definition of the “DM” group. We appreciate the opportunity to clarify as follows.

According to the ADA Standards of Care, either the 2019 version (PMID: 30559228) or the most recent 2026 update (PMID: 41358893), the diagnosis of diabetes remains unchanged and is based on four glycemic criteria: A1C ≥6.5%, FPG ≥126 mg/dL, OGTT 2h PG ≥200 mg/dL, or random PG ≥200 mg/dL with classic symptoms. These diagnostic thresholds are identical for all types of diabetes, including T1D, T2D, and gestational diabetes. Classification into T1D versus T2D is a separate step that relies on clinical features, autoantibodies, and C-peptide levels; therefore, the ADA diagnostic criteria themselves do not distinguish between T1D and T2D. Our patients were enrolled according to these ADA diagnostic criteria, and we have therefore used the term “DM” (referred to “diabetes”) without sub-classification. We acknowledge that this may have caused confusion, and we have now provided a detailed description of the ADA criteria used for diagnosing diabetes in the patient cohort under the Methods section (Page 8, Lines 122-126). In addition, we have explained in more detail which measures were used to define COPD according to the GOLD guidelines in the revised manuscript.

Regarding the HbA1c results showing a correlation between acute exacerbations (AE) and HbA1c levels, we apologize for making this point unclear in our original manuscript. Our primary intention in analyzing HbA1c (Figure EV1, Table EV1) was to provide a quantifiable clinical indicator of diabetes that correlates with AECOPD and to support the link between diabetes and COPD severity. It was not our intention to imply that our enrolled diabetic patients needed to be grouped or analyzed based on high HbA1c values (hyperglycemia, i.e. uncontrolled diabetes). Furthermore, HbA1c is highly relevant to the AGEs investigated in our subsequent studies, as it is one of the most studied and clinically utilized AGEs, formed via a nonenzymatic reaction between glucose and the amino groups of hemoglobin (PMID: 32657776). Taken together, using this marker, we observed that COPD patients with frequent acute exacerbations (AE) had higher HbA1c levels, thereby demonstrating a positive correlation between diabetes and COPD. This does not imply that our diabetic samples were derived from patients with high HbA1c values. To avoid any ambiguity, we have added the following description in the revised manuscript (Page 16, Lines 355-357) to clarify why we analyzed HbA1c: "HbA1c is a standard biomarker for monitoring diabetes severity monitoring [49]. To analyze the correlation between diabetes and COPD, we further investigated the relationship between HbA1c and frequent AE."

As you suggested, we analyzed the proportion of frequent acute exacerbations (AE) according to diabetes status (with vs. without diabetes) and according to HbA1c levels (⩾6.5% vs. <6.5%). The results (data shown in Fig. EV1 C-E) showed that the increase in the proportion of frequent AE was similar between the two grouping strategies. This suggests that both grouping methods are valid for analyzing frequent AE and are largely consistent. Moreover, this may be partly because the majority

of enrolled diabetic patients happened to have HbA1c levels above 6.5%.

Regarding your suggestion to include separate correlations for T1D and T2D, as detailed above, our enrolled cases were type 2 diabetes (T2D); no type 1 diabetes cases were present in the cohort. Consequently, we could not include separate correlations for T1D and T2D. We acknowledge this limitation and have added the statement under the Discussion section (Page 26, Lines 629-635), as stated previously.

We hope these clarifications and revisions address your concerns. Thank you again for the insightful comments.

3) The phrase "diabetic COPD patients" should not be used, rather patient with T1D/ patient with T2D

Response: Thank you for this terminological suggestion. We have clarified that all diabetic patients in our cohort had type 2 diabetes (T2D) in "Clinical data collection" and "Ethics for human sample collection" subsections under the Methods section. As noted in our response to previous comments, we have also revised the phrase "diabetic COPD patients" to "COPD patients with diabetes" throughout the manuscript. Thank you for pointing it out.

4) Authors "demonstrated significant enrichment of multiple viral infection pathways, suggesting altered antiviral defense capacity in COPD patients with DM compared with those without DM". The question is whether this increased antiviral defense observed in "DM" is really the reason for severe COPD or whether patients with T1D and T2D anyway have a basally elevated antiviral defense gene expression, also in lung epithelial, and this is independent of COPD; this needs to be checked by including control patients with "DM" without COPD.

Response: Thank you for the insightful mechanistic question. We fully agree that in order to definitively conclude that the altered antiviral defense observed in COPD patients with diabetes contributes to disease exacerbation, an ideal experimental design would include a control group of diabetic patients without COPD. We regret that we were unable to include such a cohort in the present study due to limited sample availability. This was primarily because, for ethical reasons and clinical practice, bronchoscopy is not routinely performed in diabetic patients without COPD, which greatly restricted our access to bronchial biopsy samples from this population.

However, recent reports have established that diabetes is associated with impaired basal antiviral response, rendering diabetic individuals more susceptible to respiratory viral infections. We believe this addresses your concern. Nobs et al. (*Nature*, 2023, PMID: 38093014) demonstrated that hyperglycemia induces epigenetic and immunometabolic dysfunction in lung dendritic cells, leading to compromised antiviral immunity. Similarly, Šestan et al. (*Nature Immunology*, 2024, PMID: 38811816) demonstrated that hyperglycemia compromises an IFNγ-dependent immune circuit essential for antiviral defense, offering a mechanistic explanation for why diabetic patients are more susceptible to viral infection. Furthermore, Gray et al. (*Cell Metabolism*, 2024, PMID: 39488214) showed that hyperglycemia-triggered lipid peroxidation impairs Th1 antiviral responses in type 2 diabetes. Collectively, these findings indicate that diabetes itself establishes a baseline state of impaired antiviral defense capacity, and this diabetes-induced baseline antiviral defect may also be present in the airway epithelium, leading to heightened viral susceptibility and severe COPD.

We acknowledged the lack of a "diabetes without COPD" control group as a limitation and have now added this discussion point under the Discussion section in the revised manuscript (Page 26,

Lines 635-645). We sincerely thank you again for this constructive comment, which has helped us refine our mechanistic interpretation and better articulate the translational implications of our findings.

5) Figure S3, for the cytokine PCR, please add all controls from Fig.1I (HG without virus) to see whether HG alone increases inflammation markers

Response: In Figure S3 (now Figure EV2), we have added all controls (HG without virus) and detected the expression of the indicated cytokines. We observed that HG treatment alone slightly upregulated the pro-inflammatory cytokines (TNF-α, IL-1β, IL-6) but did not alter the expression of interferon-stimulated genes (ISGs, e.g., IFNB, ISG15, OAS1, MX1). Following IAV infection post-HG treatment, both pro-inflammatory cytokines and ISGs were elevated compared to the IAV-alone group. This suggests that the increase in pro-inflammatory cytokines (TNF-α, IL-1β, IL-6) likely results from the combined effects of HG and the virus. In contrast, the upregulation of ISGs (IFNB, ISG15, OAS1, MX1) is primarily driven by the virus. Notably, the elevated expression of these genes probably reflects a compensatory host response secondary to increased viral loads, which is consistent with the concept that impaired antiviral defense leads to enhanced viral loads. We have updated the Results section accordingly with these new data.

6) From Fig.1J one cannot see increased viral replication in glucose-exposed epithelium

Response: Thank you for pointing it out. In the original Fig. 1J, we aimed to visualize viral abundance by immunofluorescence staining of the viral NS1 protein. We have performed immunofluorescence staining of NS1 protein on a total of 4 biological replicates, quantified NS1 mean fluorescence intensity (MFI), and added the statistical analysis. The results showed that NS1 staining was significantly increased in high glucose-exposed ALI cultures compared to control cultures, confirming that glucose-exposed epithelium exhibits significantly higher viral loads. Based on these findings, we have replaced the original image in Fig. 1J with a clearer representative picture to support our conclusion.

7) The blots should also show samples from "DM". What was the "DM" sample? High HbA1c?

Response: Thank you for the question. As mentioned above, “DM” referred to “diabetes” based on the ADA diagnostic criteria. The “DM” samples were bronchial biopsy specimens obtained from the airway of COPD patients with diabetes. These samples were not obtained based on high HbA1c values. To avoid any ambiguity, we have now replaced “DM” with “diabetes” throughout the manuscript and figures, and provided a detailed description of the ADA diagnostic criteria in the Methods section.

Regarding HbA1c, the data presented in Figure EV1 and Table EV1 were intended to introduce a quantifiable clinical indicator of diabetes and to analyze the correlation between diabetes and COPD. Using this marker, we observed that COPD patients with frequent acute exacerbations had higher HbA1c levels, demonstrating a positive correlation between diabetes and COPD. This does not imply that our diabetic samples were derived only from patients with high HbA1c values. We hope this clarifies the issue. Thank you again for pointing this out.

8) Blot in Fig.5B, please remove the dotted lines, as there is no lane/blot separation.

Response: We agree that the dotted lines in Fig. 5B were unnecessary because there was no lane

or blot separation. We have removed them in the revised figure. Thank you for pointing this out.

Referee #3 (Comments on Novelty/Model System for Author):
The combination of human patient data, in vitro air-liquid interface cultures, and mouse is adequate for the studies. I have suggested additional experiments to be performed with the mouse model.

Referee #3 (Remarks for Author):
Review of the EMMM-2025-23076 by Chen et al.,

Targeting Diabetes-Exacerbated COPD Progression: Blocking AGEs to Rescue Viral Susceptibility in the Airway Epithelium

Individuals with diabetes have accelerated COPD progression, but the underlying mechanisms are poorly understood. In this study, Chen et al use scRNA-seq of airway epithelium to identify transcriptional alterations in diabetic versus non-diabetic COPD patients, focusing on host-defense pathways to viral infection, a key cause of COPD exacerbations. They found that hyperglycemia enhanced influenza virus susceptibility in both COPD-derived epithelial cells and diabetic COPD mice, suggesting a compromised airway defense. Furthermore, hyperglycemia promotes advanced glycation end product (AGEs) accumulation in the airway epithelium that correlates with infection severity. AGEs activate p38/ERK/IKK proinflammatory signaling via RAGE and MD2 receptors, leading to epithelial injury and heightened viral susceptibility. Inhibition of AGEs formation with aminoguanidine significantly reduced viral load. The authors concluded that the hyperglycemia-AGEs-RAGE/MD2 axis increases viral susceptibility in diabetic COPD patients. Overall, this is an extensive characterization of the contribution of AGEs to the pathogenesis of diabetic COPD. The manuscript is well written. In general, various conclusions are supported by extensive amounts of convincing experimental data. One problem with this study is that among the diabetics, the authors did not distinguish between type 1 versus type 2 diabetes, which should be clearly indicated in patient profiles, and may help to stratify type 1 vs. type 2 associated COPD phenotypes with regard to respiratory AGEs. Also, the deleterious effects of AGEs are well known and have been widely studied. The relative importance of RAGE vs. MD2 induced by diabetic COPD AGEs needs to be established. Whether or not AGEs in diabetic COPD-derived AGEs activate the non-canonical pathway (e.g., TGFβ2, ERK, AKT, and p38 MAPK) signaling is not addressed at all. A few additional minor suggestions are provided to further improve the manuscript.

Response: We sincerely thank you for the positive and constructive evaluation of our work. We are also grateful for the thoughtful criticisms and valuable suggestions, which have helped us substantially improve the manuscript. We have carefully addressed all the points raised by you, as detailed below.

Major comments:
1. Introduction, Lines 63-66. It will be useful to briefly describe the structural and functional defects of airway epithelial cells in COPD patients that may increase susceptibility to microbial infections, including the compromised barrier function.

Response: Thank you for the suggestion. We have added a brief description with “In COPD

patients, the airway epithelium exhibits structural and functional defects, including ciliary dysfunction, mucus hypersecretion, and impaired barrier integrity, all of which may contribute to increased susceptibility to viral infections." under the Introduction section (Page 4, Lines 81-84) in the revised manuscript.

2. Material and Methods, Lines 123-130. The description provided on the diabetic COPD mouse model is not clear. Please clearly indicate that diabetic mice were treated with porcine pancreatic elastase. Since elastase treatment requires 4 weeks, how was the diabetic state maintained in these mice?

Response: Thank you for the comment. To clarify, diabetic COPD mice were generated by first injecting STZ to induce diabetic hyperglycemia, followed by intranasal instillation of elastase to induce COPD three days later. The diabetic state was maintained throughout the 4-week COPD development period, as confirmed by weekly blood glucose monitoring (data shown in Fig. EV4C). It is well established that a single high-dose STZ injection induces stable hyperglycemia lasting well beyond 4 weeks (PMID: 29154192; PMID: 35370759; PMID: 34003304). At the endpoint, successful comorbidity was validated by persistent hyperglycemia (≥15 mM) and emphysematous changes (enlarged alveoli, increased mean linear intercept). Importantly, we detected significantly increased AGEs accumulation in the airways of these mice, recapitulating the clinical observation of AGEs deposition in the airway epithelium of COPD patients with diabetes (data shown in Fig. 3E-F). This supports the relevance of our mouse model for investigating diabetes-exacerbated susceptibility to viral infection. We apologize for the lack of clarity and have revised the Methods section accordingly to provide more detail.

3. Results, triplicate of histopathology, immunofluorescence images of ALI culture labeling, and western blots, as well as their quantification (e.g., with ImageJ, etc), need to be provided for all figures.

Response: We have provided triplicate representative images for histopathology, immunofluorescence images of ALI culture labeling, and western blots in the Source Data files of each figure. Quantifications are presented alongside the representative images where applicable. For panels that are for qualitative purposes (e.g., morphological assessment or subcellular localization), we have clarified in the figure legends with the description "Representative images from three independent experiments are shown." accordingly. Thank you for this suggestion.

4. Results, Table S1. A major problem with this study is that among the diabetics, the authors did not distinguish between type 1 versus type 2 diabetes, which should be clearly indicated in patient profiles in Table S1, and may help to stratify type 1 vs. type 2 associated COPD phenotypes with regard to respiratory AGEs.

Response: We thank you for this important comment. In response, we have now added a new column "Diabetes type" in Table S1 (now Table EV1). After re-examining the clinical information, we found that all identified diabetic cases were type 2 diabetes (T2D); no type 1 diabetes (T1D) cases were present in the cohort. We fully agree with your point that distinguishing between T1D and T2D is important for phenotypic stratification. However, given the absence of T1D patients in our current cohort, we could not perform separate analyses by diabetes type. We acknowledge this limitation and we have explicitly stated this limitation in the Discussion section (Page 26, Lines

629-635) with the description "all diabetic patients in our cohort had T2D, with no T1D cases identified; therefore, we did not stratify our analysis by diabetes type. Nevertheless, although the etiologies of T1D and T2D differ, both conditions converge on chronic hyperglycemia, which is the key driver of diabetic complications. Moreover, AGEs, the central molecule of this study and a key downstream product of hyperglycemia, are elevated in both types of diabetes [71-72], suggesting that similar findings may occur in both T1D and T2D populations."

We hope these clarifications and revisions address your concerns. Thank you again for the insightful comments, which have helped us to more clearly define the scope and limitations of our study.

5. Fig. 1G-1J. 28 days are inadequate to allow full differentiation of ALI culture, where ~42 days are typically needed.

Response: Thank you for this suggestion. However, we would like to respectfully point out that the timeframe for achieving full differentiation in Air-Liquid Interface (ALI) cultures is not uniform across all cell types and experimental systems. While some protocols, particularly those using primary cells from certain species or requiring extensive ciliogenesis and mucus production, may indeed benefit from extended culture periods, a substantial body of literature supports the use of a 28-day differentiation protocol as sufficient for establishing a fully differentiated, pseudostratified mucociliary epithelium of the respiratory tract.

For instance, Goldfarbmuren et al. (PMID: 32427931) defined successful ALI culture as one in which a pseudostratified epithelium has formed after 21 days in the presence of all differentiated epithelial cell types. Similarly, a study by Swart et al. (PMID: 38858595) successfully differentiated primary human bronchial epithelial cells at ALI for 25 days and observed robust pseudostratified mucociliary differentiation. Furthermore, our own group has consistently validated this 28-day differentiation window in our previous publications (PMID: 37301869; PMID: 39033934). In these studies, we have demonstrated that by day 28 post-ALI establishment, the airway epithelial cultures exhibit typical morphological characteristics of a fully differentiated airway epithelium, including the presence of ciliated cells, goblet cells, and basal cells, as well as functional barrier integrity. These findings confirm that a 28-day ALI culture is entirely adequate for our experimental model.

We appreciate your attention to this methodological detail and we have revised the manuscript to cite these supporting references, thereby reinforcing the robustness of our differentiation protocol.

6. Figs. S3, S4, S5, 4G, and 5C. ELISA assays should be performed to support the qPCR-based expression of cytokines and anti-viral genes.

Response: We have performed ELISA assays and the ELISA results were consistent with the qPCR findings for both cytokines (e.g., TNFα, IL-1β, IL-6) and antiviral genes (e.g., IFNB). These data have been added to the revised manuscript accordingly. Thank you for pointing it out.

7. Fig. 5. The studies with RAGE and MD2 inhibitors and their impact on the expression of AGEs, anti-viral genes and cytokines, and viral loads need to be authenticated in the diabetic COPD mice.

Response: We thank you for this valuable suggestion. Accordingly, we performed the requested animal experiments to authenticate the impact of RAGE and MD2 inhibitors in diabetic COPD mice (data shown in Fig. EV5). Our new results demonstrate that treatment with RAGE or MD2 inhibitors significantly attenuated viral loads, reduced pro-inflammatory signaling activation, and suppressed

the expression of anti-viral genes and pro-inflammatory cytokines in diabetic COPD mice, consistent with the results obtained from *in vitro* ALI cultures. These findings have been added in the Results section in the revised manuscript.

8. In addition to inhibitors, the importance of RAGE and MD2 induced by diabetic COPD AGEs needs to be confirmed with siRNA studies.

Response: We have performed siRNA knockdown experiments as requested (data presented in Fig. 5). The results show that RAGE or MD2 knockdown reduced AGE-induced pro-inflammatory signaling activation, airway epithelial injury, and viral susceptibility, which is consistent with and further confirmed by our inhibitor data. Thank you for this important suggestion.

9. The relative importance of RAGE vs. MD2 induced by diabetic COPD AGEs needs to be established.

Response: Thank you for this insightful point. To establish the relative importance of RAGE versus MD2 induced by diabetic COPD AGEs, we performed inhibitor and siRNA knockdown experiments targeting RAGE or MD2 individually, as well as their combination (data presented in Fig. 5). The results showed that inhibition or knockdown of RAGE alone significantly reduced AGE-induced pro-inflammatory cytokine expression, epithelial cell injury, and susceptibility to IAV infection, whereas inhibition or knockdown of MD2 alone also exerted inhibitory effects but to a notably lesser extent, and combined inhibition or knockdown of both RAGE and MD2 appears to result in greater protection than either intervention alone. These findings suggest that, in the context of diabetic COPD, RAGE may play a more dominant role than MD2 in mediating the pathological effects triggered by AGEs. However, both receptors contribute to the process, and their combined targeting appears to yield additive/synergistic effects. Accordingly, we have added these descriptions under the Results section in the revised manuscript (Page 22, Lines 538-543).

10. How AGEs in diabetic COPD-derived AGEs in activating the non-canonical pathway (e.g., TGFβ2, ERK, AKT, and p38 MAPK) signaling is not addressed at all.

Response: Thank you for raising this important point. We have performed experiments to examine the activation status of TGFβ2, ERK, AKT, p38 MAPK, and IKK following AGEs exposure (data shown in Fig. 4F). Our results show that: ERK, p38 MAPK and IKK were activated by AGEs, as indicated by increased levels of phospho‑ERK, phospho‑p38 and phospho‑IKK. In contrast, TGFβ2 and AKT were not significantly activated under the same experimental conditions (no increase in phospho-smad2/3 or phospho-AKT). These findings indicate that AGEs selectively activate certain pathways (ERK, p38 MAPK and IKK) but not others (TGFβ2 and AKT) in our airway epithelial model.

In Fig. 5, we further validated that AGEs activate the ERK, p38 MAPK, and IKK pathways through RAGE and MD2, which are receptors for AGEs. Actually, it has been documented that RAGE and MD2 can activate the ERK, p38 MAPK, and IKK pathways. Mechanistically, Yan et al. (PMID: 33436632) reported that SLP76 serves as an adaptor protein that binds to the cytosolic tail of RAGE, thereby activating the p38 MAPK, ERK1/2, and IKKα/β pathways. Wang et al. (PMID: 28045026; PMID: 32358497) demonstrated that the TLR4/MD2 complex, upon activation by ligands such as saturated palmitic acid or high glucose, triggers the activation of ERK, p38, and IKK/NF-κB signaling pathways. Therefore, our observation that ERK, p38 MAPK, and IKK are

common downstream effectors of both receptors is consistent with the literature. Accordingly, we have also added a brief discussion on the potential implications of this activation under the Discussion section (Page 25, Lines 608-613). in the revised manuscript.

Minor comments

1. Introduction. Lines 85-86. ALI culture should be classified as in vitro, not ex vivo, which is typically reserved for organ cultures such as precision-cut lung slices.

Response: Thank you for this terminological clarification. We agree that air-liquid interface (ALI) cultures are established from dissociated primary epithelial cells and therefore should be classified as an *in vitro* model, not *ex vivo*. We have corrected this terminology throughout the text where applicable in the revised manuscript. We apologize for this inaccurate wording.

2. Materials and Methods. Lines 109-115. The two sentences here sound redundant and need to be combined and rephrased.

Response: We have combined and rephrased the two sentences as follows: "Human lung tissues, bronchial brushings, and bronchoalveolar lavage fluid (BALF) from COPD patients with or without diabetes were obtained from The First Affiliated Hospital of Guangzhou Medical University. The study protocols were approved by the hospital's ethics committee in conformity with the Declaration of Helsinki (approval numbers: 2018-92, 2025-080-02)." This revised version has been incorporated into the Materials and Methods section in the revised manuscript. Thank you for the suggestion.

3. Results, Lines 178-179. Please rephrase. The epithelial barrier is one of the primary airway defenses, but there are many other important ones, such as alveolar macrophages, surfactant lipid and proteins, antimicrobial peptides, etc.

Response: We have revised the sentence to "Frequent AE in COPD are closely associated with respiratory viral and bacterial infections, where the airway epithelial barrier serves as one of the primary defenses." This change acknowledges that the epithelial barrier is among multiple airway defense mechanisms. Thank you for pointing it out.

4. Fig. S8, please define PPE and STZ in the legend.

Response: We have now defined "PPE" as porcine pancreatic elastase and "STZ" as streptozotocin in the legend of Fig. S8 (now Fig. EV4). The revised legend has been updated in the manuscript.

5. Lines 303-304. Please provide references for AGEs interaction with RAGE and MD2.

Response: Thank you for pointing it out. We have incorporated the references that document the interaction of AGEs with RAGE and MD2.

17th Jun 2026

Dear Dr. Peng,

Thank you for submitting your revised manuscript. Referees #1 and #3 have now evaluated the revised version of your study. As you will see below, the referees are satisfied with the revisions, and I will therefore be able to accept your manuscript once the following points have been addressed:

1/ Manuscript text:
- Please remove the red font text and only keep in track changes mode any new modification.
- Discrepancies were found in the following name: Deyun Wang (manuscript) vs De Yun Wang (submission system), please correct. Please add the institutional email addresses for corresponding authors Yang Peng, Ziqing Zhou and Dr. Lei Wang to the manuscript title page. They can be added in addition to the authors' personal email addresses and are already present in our system.
- Please note that all corresponding authors are required to supply an ORCID ID for their name upon submission of a revised manuscript. ORCID identifiers are missing for Lei Wang, Ziqing Zhou and Panpan Hou.
- Please place the Methods Section after the Discussion. Remove the reagent table from the manuscript text and upload it as an independent file.
- In the methods:
o Human samples: please include a statement confirming that informed consent was obtained from all subjects, and the full statement that the experiments conformed to the principles set out in the WMA Declaration of Helsinki and the Department of Health and Human Services Belmont Report.
o Mice experiments: please provide the origin of the mice.
o Antibody: please provide dilutions/concentrations.
o SEM section: please check the language.
o Statistics: please provide a statement on exclusion/inclusion criteria, blinding and randomization.
- Data Availability: Please note that the specific URL for PRJCA063746 dataset is not provided in the data availability statement. Datasets must be publicly available before acceptance of the manuscript.
- Acknowledgements: only the National Natural Science Foundation of China grants are in our system (82301273, 82501001, 82270001, 82370148, 82301945); please ensure that all funding sources are listed in our system in addition to the Acknowledgements, as they will be linked in PubMed.
- "Conflict of interest" should be renamed "Disclosure and Competing Interests Statement".
- Remove the authors contributions from the manuscript text, and provide CRediT (Contributor Role Taxonomy) terms in the submission system instead.
- References should be corrected to alphabetical order, with 10 author names listed before et al.

2/ Figures:
- Please remove the titles from the figure files
- The four EV tables should be uploaded as four separate files.
- Please note that we can accommodate more than five Expanded View (EV) figures. You are therefore welcome to convert your current Appendix figures into EV figures to improve accessibility for readers. If you choose to keep an Appendix, please include page numbers in the table of contents.
- Please ensure that the EV tables are cited in correct sequential order when the Methods have been moved after the Discussion section.
- Please address the queries from our data editors in the figure legends:
1. Please note that the exact p values are not provided in the legends of figures 1H-J; 2C, D, F, G, I, J, K-M; 3B, C, E, F, H, J, K, L; 4F, G; 5B, C, D, E, G, H, I, J; EV2A-D, F, G; EV3 B, D, I; EV4 B, C; EV5 A-E.
2. Please note that the yellow dashed borders are not defined in the legend of figure EV3 B. This needs to be rectified.

3/ Checklist:
- fill in all subsections of "experimental study design and statistics".
- fill in "Data Availability", subsection "Were human clinical and genomic datasets deposited in a public access-controlled repository in accordance to ethical obligations to the patients and to the applicable consent agreement".

4/ Synopsis:
Please upload a visual abstract without legend, in jpeg, tiff or png format, with the exact dimensions 550 pixels wide x 300 pixels high. The image will need to be simplified to fit these dimensions and remain legible.

5/ As part of the EMBO Publications transparent editorial process initiative (see our Editorial at http://embomolmed.embopress.org/content/2/9/329), EMBO Molecular Medicine will publish online a Review Process File (RPF) to accompany accepted manuscripts.

This file will be published in conjunction with your paper and will include the anonymous referee reports, your point-by-point response and all pertinent correspondence relating to the manuscript. Let us know whether you agree with the publication of the RPF.
Please note that the Authors checklist will be published at the end of the RPF.

I look forward to receiving your revised manuscript.

Yours sincerely,

Lise Roth

Lise Roth, PhD
Senior Editor
EMBO Molecular Medicine

***** Reviewer's comments *****

Referee #1 (Comments on Novelty/Model System for Author):

All previous suggestions addressed and manuscript well improved

Referee #1 (Remarks for Author):

Congratulations to an important study!

Referee #3 (Comments on Novelty/Model System for Author):

The models are adequate.

Referee #3 (Remarks for Author):

This is a markedly improved manuscript. The authors have really going out of their way to address all queries raised by reviewers.

The authors addressed the remaining editorial isues.

7th Jul 2026

Dear Dr. Peng,

Thank you for submitting your revised files. I am pleased to inform you that your manuscript is accepted for publication and is now being sent to our publisher to be included in the next available issue of EMBO Molecular Medicine.

Please note that, as discussed in a previous correspondence, I have changed the data availability section to: "The single cell sequencing data produced in this study have been deposited in the SRA database, under accession number PRJNA1490316 (https://www.ncbi.nlm.nih.gov/sra/PRJNA1490316).
Informed consent was obtained for data deposition and sharing, and the data have been deposited in accordance with the relevant ethical approvals and applicable privacy."

Your manuscript will be processed for publication by EMBO Press. It will be copy edited and you will receive page proofs prior to publication. Please note that you will be contacted by Springer Nature Author Services to complete licensing and payment information.

You may qualify for financial assistance for your publication charges - either via a Springer Nature fully open access agreement or an EMBO initiative. Check your eligibility: https://link.springer.com/journal/44321/how-to-publish-with-us

Should you be planning a Press Release on your article, please notify the production team via this form in order to coordinate publication and release dates: https://forms.cloud.microsoft/e/eH4A8JVP6g

If you have any questions, please do not hesitate to contact the Editorial Office. Thank you for your contribution to EMBO Molecular Medicine.

Yours sincerely,

Lise Roth

Lise Roth, Ph.D
Senior Editor
EMBO Molecular Medicine

>>> Please note that it is EMBO Molecular Medicine policy for the transcript of the editorial process (containing referee reports and your response letter) to be published as an online supplement to each paper. If you do NOT want this, you will need to inform the Editorial Office via email immediately. More information is available here: https://link.springer.com/partners/embo-press/editorial-policies#Peer%20review

EMBO Press Author Checklist

Corresponding Author Name: Panpan Hou, Ziqing Zhou, Lei Wang, Yang Peng	
Journal Submitted to: EMBO Molecular Medicine	
Manuscript Number: EMM-2025-23076	

USEFUL LINKS FOR COMPLETING THIS FORM
The EMBO Journal - Author Guidelines
EMBO Reports - Author Guidelines
Molecular Systems Biology - Author Guidelines
EMBO Molecular Medicine - Author Guidelines

Reporting Checklist for Life Science Articles (updated January
This checklist is adapted from Materials Design Analysis Reporting (MDAR) Checklist for Authors. MDAR establishes a minimum set of requirements in transparent reporting in the life sciences (see Statement of Task: 10.31222/osf.io/9sm4x). Please follow the journal's guidelines in preparing your
Please note that a copy of this checklist will be published alongside your article.

Abridged guidelines for figures
1. Data
The data shown in figures should satisfy the following conditions:
- the data were obtained and processed according to the field's best practice and are presented to reflect the results of the experiments in an accurate and unbiased manner.
- ideally, figure panels should include only measurements that are directly comparable to each other and obtained with the same assay.
- plots include clearly labeled error bars for independent experiments and sample sizes. Unless justified, error bars should not be shown for technical
- if n<5, the individual data points from each experiment should be plotted. Any statistical test employed should be justified.
- Source Data should be included to report the data underlying figures according to the guidelines set out in the authorship guidelines on Data

2. Captions
Each figure caption should contain the following information, for each panel where they are relevant:
- a specification of the experimental system investigated (eg cell line, species name).
- the assay(s) and method(s) used to carry out the reported observations and measurements.
- an explicit mention of the biological and chemical entity(ies) that are being measured.
- an explicit mention of the biological and chemical entity(ies) that are altered/varied/perturbed in a controlled manner.
- the exact sample size (n) for each experimental group/condition, given as a number, not a range;
- a description of the sample collection allowing the reader to understand whether the samples represent technical or biological replicates (including how many animals, litters, cultures, etc.).
- a statement of how many times the experiment shown was independently replicated in the laboratory.
- definitions of statistical methods and measures:
  - common tests, such as t-test (please specify whether paired vs. unpaired), simple χ2 tests, Wilcoxon and Mann-Whitney tests, can be unambiguously identified by name only, but more complex techniques should be described in the methods section;
  - are tests one-sided or two-sided?
  - are there adjustments for multiple comparisons?
  - exact statistical test results, e.g., P values = x but not P values < x;
  - definition of 'center values' as median or average;
  - definition of error bars as s.d. or s.e.m.

Please complete ALL of the questions below.
Select "Not Applicable" only when the requested information is not relevant for your study.

Materials

Newly Created Materials	Information included in the manuscript?	In which section is the information available? (Reagents and Tools Table, Materials and Methods, Figures, Data Availability Section)
New materials and reagents need to be available; do any restrictions apply?	Not Applicable	

Antibodies	Information included in the manuscript?	In which section is the information available? (Reagents and Tools Table, Materials and Methods, Figures, Data Availability Section)
For **antibodies** provide the following information:<br>- Commercial antibodies: RRID (if possible) or supplier name, catalogue number and or/clone number<br>- Non-commercial: RRID or citation	Yes	Reagents and Tools Table

DNA and RNA sequences	Information included in the manuscript?	In which section is the information available? (Reagents and Tools Table, Materials and Methods, Figures, Data Availability Section)
**Short novel DNA or RNA including primers, probes**: provide the sequences.	Yes	Methods, Table EV4

Cell materials	Information included in the manuscript?	In which section is the information available? (Reagents and Tools Table, Materials and Methods, Figures, Data Availability Section)
**Cell lines:** Provide species information, strain. Provide accession number in repository **OR** supplier name, catalog number, clone number, and/**OR** RRID.	Not Applicable	
**Primary cultures:** Provide species, strain, sex of origin, genetic modification status.	Yes	Methods
Report if the cell lines were recently **authenticated** (e.g., by STR profiling) and tested for mycoplasma contamination.	Not Applicable	

Experimental animals	Information included in the manuscript?	In which section is the information available? (Reagents and Tools Table, Materials and Methods, Figures, Data Availability Section)
**Laboratory animals or Model organisms:** Provide species, strain, sex, age, genetic modification status. Provide accession number in repository **OR** supplier name, catalog number, clone number, **OR** RRID.	Yes	Methods
**Animal observed in or captured from the field:** Provide species, sex, and age where possible.	Not Applicable	
Please detail **housing and husbandry conditions**.	Yes	Methods

Plants and microbes	Information included in the manuscript?	In which section is the information available? (Reagents and Tools Table, Materials and Methods, Figures, Data Availability Section)
**Plants:** provide species and strain, ecotype and cultivar where relevant, unique accession number if available, and source (including location for collected wild specimens).	Not Applicable	
**Microbes:** provide species and strain, unique accession number if available, and source.	Yes	Methods

Human research participants	Information included in the manuscript?	In which section is the information available? (Reagents and Tools Table, Materials and Methods, Figures, Data Availability Section)
If collected and within the bounds of privacy constraints report on age, sex and gender or ethnicity for all study participants.	Yes	Methods

Core facilities	Information included in the manuscript?	In which section is the information available? (Reagents and Tools Table, Materials and Methods, Figures, Data Availability Section)
If your work benefited from core facilities, was their service mentioned in the acknowledgments section?	Yes	Acknowledgements

Design

Study protocol	Information included in the manuscript?	In which section is the information available? (Reagents and Tools Table, Materials and Methods, Figures, Data Availability Section)
If study protocol has been **pre-registered, provide DOI in the manuscript**. For clinical trials, provide the trial registration number **OR** cite DOI.	Not Applicable	
Report the **clinical trial registration number** (at ClinicalTrials.gov or equivalent), where applicable.	Not Applicable	

Laboratory protocol	Information included in the manuscript?	In which section is the information available? (Reagents and Tools Table, Materials and Methods, Figures, Data Availability Section)
Provide DOI OR other citation details if **external detailed step-by-step protocols** are available.	Not Applicable	

Experimental study design and statistics	Information included in the manuscript?	In which section is the information available? (Reagents and Tools Table, Materials and Methods, Figures, Data Availability Section)
Include a statement about **sample size** estimate even if no statistical methods were used.	Yes	Methods
Were any steps taken to minimize the effects of subjective bias when allocating animals/samples to treatment (e.g. **randomization procedure**)? If yes, have they been described?	Yes	Methods
Include a statement about **blinding** even if no blinding was done.	Yes	Methods
Describe **inclusion/exclusion criteria** if samples or animals were excluded from the analysis. Were the criteria pre-established?<br><br>If sample or data points were omitted from analysis, report if this was due to attrition or intentional exclusion and provide justification.	Yes	Methods
For every figure, are **statistical tests** justified as appropriate? Do the data meet the assumptions of the tests (e.g., normal distribution)? Describe any methods used to assess it. Is there an estimate of variation within each group of data? Is the variance similar between the groups that are being statistically compared?	Yes	Methods, Figure legend

Sample definition and in-laboratory replication	Information included in the manuscript?	In which section is the information available? (Reagents and Tools Table, Materials and Methods, Figures, Data Availability Section)
In the figure legends: state number of times the experiment was **replicated** in laboratory.	Yes	Figure legend
In the figure legends: define whether data describe **technical or biological replicates**.	Yes	Figure legend

Ethics

Ethics	Information included in the manuscript?	In which section is the information available? (Reagents and Tools Table, Materials and Methods, Figures, Data Availability Section)
Studies involving **human participants**: State details of **authority granting ethics approval** (IRB or equivalent committee(s), provide reference number for approval.	Yes	Methods
Studies involving **human participants**: Include a statement confirming that **informed consent** was obtained from all subjects and that the experiments conformed to the principles set out in the WMA Declaration of Helsinki and the Department of Health and Human Services Belmont Report.	Yes	Methods
Studies involving **human participants: For publication of patient photos**, include a statement confirming that consent to publish was obtained.	Not Applicable	
Studies involving experimental **animals**: State details of **authority granting ethics approval** (IRB or equivalent committee(s), provide reference number for approval. Include a statement of compliance with ethical regulations.	Yes	Methods
Studies involving **specimen and field samples:** State if relevant **permits** obtained, provide details of authority approving study; if none were required, explain why.	Not Applicable	

Dual Use Research of Concern (DURC)	Information included in the manuscript?	In which section is the information available? (Reagents and Tools Table, Materials and Methods, Figures, Data Availability Section)
Could your study fall under dual use research restrictions? Please check biosecurity documents and list of **select agents and toxins** (CDC): https://www.selectagents.gov/sat/list.htm	Not Applicable	
If you used a select agent, is the security level of the lab appropriate and reported in the manuscript?	Not Applicable	
If a study is subject to dual use research of concern regulations, is the name of the **authority granting approval and reference number** for the regulatory approval provided in the manuscript?	Not Applicable	

Reporting

The MDAR framework recommends adoption of discipline-specific guidelines, established and endorsed through community initiatives. Journals have their own policy about requiring specific guidelines and recommendations to complement MDAR.

Adherence to community standards	Information included in the manuscript?	In which section is the information available? (Reagents and Tools Table, Materials and Methods, Figures, Data Availability Section)
State if relevant guidelines or checklists (e.g., **ICMJE, MIBBI, ARRIVE, PRISMA**) have been followed or provided.	Not Applicable	
For **tumor marker prognostic studies,** we recommend that you follow the **REMARK** reporting guidelines (see link list at top right). See author guidelines, under 'Reporting Guidelines'. Please confirm you have followed these guidelines.	Not Applicable	
For **phase II and III randomized controlled trials**, please refer to the **CONSORT** flow diagram (see link list at top right) and submit the CONSORT checklist (see link list at top right) with your submission. See author guidelines, under 'Reporting Guidelines'. Please confirm you have submitted this list.	Not Applicable	

Data Availability

Data availability	Information included in the manuscript?	In which section is the information available? (Reagents and Tools Table, Materials and Methods, Figures, Data Availability Section)
Have **primary datasets** been deposited according to the journal's guidelines (see 'Data Deposition' section) and the respective accession numbers provided in the Data Availability Section?	Yes	Data availability
Were **human clinical and genomic datasets** deposited in a public access-controlled repository in accordance to ethical obligations to the patients and to the applicable consent agreement?	Yes	Methods
Are **computational models** that are central and integral to a study available without restrictions in a machine-readable form? Were the relevant accession numbers or links provided?	Not Applicable	
If publicly available data were reused, provide the respective **data citations in the reference list**.	Not Applicable	
